# Unraveling the occasional occurrence of berry astringency in table grape cv. Scarlet Royal: a physiological and transcriptomic analysis

**DOI:** 10.3389/fpls.2023.1271251

**Published:** 2023-10-26

**Authors:** Ahmed Ismail, Tariq Pervaiz, Stacey Comstock, Sohrab Bodaghi, Alaaeldin Rezk, Georgios Vidalakis, Islam El-Sharkawy, David Obenland, Ashraf El-kereamy

**Affiliations:** ^1^ Department of Botany and Plant Sciences, University of California Riverside, Riverside, Riverside, CA, United States; ^2^ Department of Horticulture, Faculty of Agriculture, Damanhour University, Damanhour, Egypt; ^3^ Department of Microbiology & Plant Pathology, University of California Riverside, Riverside, Riverside, CA, United States; ^4^ Center for Viticulture and Small Fruit Research, College of Agriculture and Food Sciences, Florida A&M University, Tallahassee, FL, United States; ^5^ United States Department of Agriculture (USDA), Agricultural Research Service, San Joaquin Valley Agricultural Sciences Center, Parlier, CA, United States

**Keywords:** berry quality, polyphenols, tannins, table grapes, gene expression, grapevine nutrients

## Abstract

Scarlet Royal, a mid-season ripening table grape, is one of the popular red grape varieties in California. However, its berries develop an undesirable astringent taste under certain conditions. Among the various factors contributing to the degradation of berry attributes, the levels and compositions of polyphenols play a fundamental role in defining berry quality and sensory characteristics. To comprehend the underlying mechanism of astringency development, Scarlet Royal berries with non-astringent attributes at the V7 vineyard were compared to astringent ones at the V9 vineyard. Biochemical analysis revealed that the divergence in berry astringency stemmed from alterations in its polyphenol composition, particularly tannins, during the late ripening stage at the V9 vineyard. Furthermore, transcriptomic profiling of berries positively associated nineteen flavonoid/proanthocyanidins (PAs) structural genes with the accumulation of PAs in V9 berries. The identification of these genes holds significance for table grape genetic improvement programs. At a practical level, the correlation between the taste panel and tannin content revealed a threshold level of tannins causing an astringent taste at approximately 400 mg/L. Additionally, berry astringency at the V9 vineyard was linked to a lower number of clusters and yield during the two study seasons, 2016 and 2017. Furthermore, petiole nutrient analysis at bloom showed differences in nutrient levels between the two vineyards, including higher levels of nitrogen and potassium in V9 vines compared to V7. It’s worth noting that V9 berries at harvest displayed a lower level of total soluble solids and higher titratable acidity compared to V7 berries. In conclusion, our results indicate that the accumulation of tannins in berries during the ripening process results in a reduction in their red color intensity but significantly increases the astringency taste, thereby degrading the berry quality attributes. This study also highlights the association of high nitrogen nutrient levels and a lower crop load with berry astringency in table grapes, paving the way for further research in this area

## 1 Introduction

Bunch grapes (*Vitis*), notably European (*Vitis vinifera*), are considered among the major fruit crops worldwide, producing roughly 70–80 million tons each year (https://www.fao.org/faostat/). Cultivars of *V. vinifera L*. are used for wine, juice, and table grape production. Grape berries are classified as non-climacteric fruits, exhibiting a double-sigmoid developmental pattern with two rapid growth phases: the berry formation (stage I) and the ripening phase (stage III), separated by an intermediate lag phase (stage II) called the green plateau (Coombe and McCarthy, 2000). The exponential increase in berry size characterizes both growth stages (I and III), but not the lag one (II). During phases (I) and (II), also known as immature stages, organic acids, mainly tartrate and malate, accumulate leading to induction of acidity levels (Kliewer, 1965; Sweetman et al., 2012; Villano et al., 2023). At the end of the lag phase, a step-change point takes place known as véraison, where acidity starts to decline while sugars, mostly glucose and fructose, as well as anthocyanins in colored varieties, increase.

Of particular interest are phenolic compounds, which are major and ubiquitous plant secondary metabolites derived from the shikimate/phenylpropanoid and polyketide pathways, with three utmost categories: (1) proanthocyanidins (PAs), also known as condensed tannins, (2) the gallo- and ellagitannins (hydrolysable tannins), and (3) the phlorotannins (Quideau et al., 2011). Such diversity of polyphenols, with more than 8000 structural variants, bestows them a wide range of biological functions ranging from growth, development, and protection inside the plant to, to some extent, human-related issues (Tresserra-Rimbau et al., 2018; Hano and Tungmunnithum, 2020). In grapevines, the accumulation pattern of phenolic compounds, along with the aforementioned berry attributes, distinguishes each of the berry phases throughout berry development (Coombe and McCarthy, 2000; Darwish et al., 2021). Indeed, berry quality and sensory characteristics (e.g., texture, flavor, and coloration) are notably defined by its polyphenol content (Gomez-Plaza et al., 2001; Duan et al., 2019; Ismail et al., 2022a). Remarkably, astringency is among the hardest sensory traits to depict and interpret as many intricate processes underpinning its perception (Ployon et al., 2018). For instance, a sensory characterisation of the astringency of 11 varietals of Italian red wine revealed that neither total phenols nor PAs can predict how all astringency subtleties will be perceived (Piombino et al., 2020). It is worth noting that the amounts, compositions, and proportions of polyphenols in a given species may vary widely depending on several factors, such as genotypic variations, developmental stages, and environmental circumstances (Mykhailenko et al., 2020; Brahmi et al., 2022).

Scarlet Royal (US Plant Patent 16229 P2) is a mid-season ripening table grape (*Vitis vinifera L*.) variety, producing seedless, red-skinned, oval-shaped, firm, and moderate to large berries with a sweet to neutral flavor (Ramming and Tarailo, 2006; Hashim-Buckey and Ramming, 2008). In the San Joaquin Valley, California, it typically ripens in mid to late August, filling the harvest window between Flame Seedless and Crimson Seedless, and has thus become a very popular red table grape variety in California. However, an undesirable astringent taste has been observed occasionally in some cases. In fact, the economic value of grapevines depends substantially on the environmental conditions, including climate, soil, cultural practices, cultivar, and rootstock. Hence, the term “terroir” is used in viticulture to describe the effect of such an interactive ecosystem on grapevine and wine quality (Van Leeuwen and Seguin, 2006).

The current study aimed to understand the underlying mechanism of astringency development in Scarlet Royal berries at two contrasting vineyards (V7 and V9). The first location (V7) produces well-colored, non-astringent berries; however, the second site (V9) yields astringent taste, poorly colored berries (hereafter referred to as V7-berries and V9-berries). The data showed a large variation in berry astringency within the same vineyard and from year to year. The data illustrated that the divergence in berry astringency stemmed from alterations in its polyphenol composition (tannins, catechin, quercetin glycosides, and total anthocyanins TAC), most notably tannins. Additionally, the ripening stage was the most distinguishing platform for such variation between both vineyards. We were able to determine the tannins’ threshold level that causes the Scarlet Royal astringency taste to be ~ 400 mg/L. Given the changes in the levels of polyphenols during berry ripening, the question was raised: what is the mechanism governing the distinctive tannins accumulation pattern between V7-berries and V9-berries, and hence astringency diversity? To answer this question, RNA-seq data generated at one ripening timepoint was associated to the changes in polyphenolic levels using a systems biology approach, WGCNA (weighted correlation network analysis). The module-trait association analysis positively correlated the key flavonoid/PAs biosynthetic genes (e.g., the genes encoding key enzymes for hydroxylation, methylation, and glycosylation) with the accumulation of tannins, catechin, and quercetin glycosides exclusively in V9-berries. The modulation of the berry’s transcriptomic profile is concomitant with its polyphenols’ composition, which finally disturbs berry quality, including astringency levels.

## 2 Materials and methods

### 2.1 Site selection and sampling

Five-year-old *V. vinifera* cv. Scarlet Royal grafted on Freedom rootstock was chosen for its berry astringency diversity at two commercial vineyards (V7 and V9) located in Delano, San Joaquin Valley, California, USA. Vineyards were located at a close distance of 10 km, and the local weather conditions during the two seasons were collected from the Delano CIMIS weather station (https://cimis.water.ca.gov/).

Both vineyards were planted at the spacing of 2.44 and 3.66 m in an open gable trellis supporting system with East-West row orientation. Vines were pruned in a Quadrilateral cordon training (spur-pruning) with 7–8 spurs (2 buds’ length) left on each cordon during the winter pruning. In addition, general UC guidelines practices were applied in both vineyard. Random forty vines from different four rows (ten from each row) from each vineyard were used in this study. Starting from veraison and until the end of the season, during two consecutive years (2016-2017). During the first year, sampling dates were July 8^th^ (S1), August 1^st^ (S2), August 10^th^ (S3), September 9^th^ (S4), September 15^th^ (S5), and October 19^th^ (S6); and for the second year, sampling dates were: July 15^th^ (S1), August 10^th^ (S2), August 25^th^ (S3), September 10^th^ (S4), September 29^th^ (S5), and October 21^st^ (S6). Sampling dates varied from the first to the second year due to the vineyard’s accessibility. At each sampling point, two sets of fifty berries were collected periodically. The first set was used to measure the berry weight, and then these berries were macerated in an electric blender, filtered through a paper towel, and an aliquot of juice was used to determine soluble solids (°Brix), pH, and titratable acidity (TA). Soluble solids were determined using a tabletop Milwaukee MA871-BOX digital refractometer (Milwaukee Instruments, Inc., NC, USA). The TA and pH were determined by titrating a 40 mL aliquot of juice with 0.1 N NaOH to a pH of 8.2 using an automatic titrator Excellence T5 (Mettler-Toledo, OH, USA). Another random 50 berries from each replicate were collected for color, tannins, and phenolic compounds and sent immediately in a cooler to EST laboratories. At harvest, which was during the month of September, an extra set of samples was collected and promptly frozen in liquid nitrogen and stored at −80°C for subsequent analysis, including RNA extraction and gene expression studies. Harvest time was determined by the growers, and the marketable clusters were picked based on the color, and yield was determined from the three harvest dates.

### 2.2 Petiole and soil nutrient analysis

At bloom, fifty leaves from each replicate were collected, resulting in a total of 200 leaves from each vineyard, for nutrient analysis. The leaf positioned at the front of the cluster was specifically selected, and the petiole was immediately separated from the blade. The petioles were transported to the laboratory, where they were triple-washed with distilled water to remove any impurities before being sent to a private laboratory for nutrient analysis. In the winter, soil samples were collected at a depth of 30 cm and at a distance of 30 cm from the vine. These samples were transported immediately to the laboratory for analysis. The nutrient content was determined using the methods described in US Salinity Laboratory Staff (1954).

### 2.3 The panel test and phenolic compounds analysis

The taste panel evaluation of Scarlet Royal table grapes was conducted with the participation of twelve nonprofessional panelists. Astringent taste perception was assessed using a scale ranging from one, representing an extremely low level of astringency, to seven, indicating an extremely high level of astringency. The taste evaluation was performed on 24 clusters from each vineyard. Phenolic compounds analysis. Total phenolic analysis was performed on 250 grams of whole berries by ETS laboratory (https://www.etslabs.com) using a reversed-phase HPLC method adapted from Price et al. (1995).

### 2.4 Nucleic acid extraction and RNA-seq library construction

Total RNA was isolated from whole berry samples following the protocol described by Boss et al. (1996). To remove any residual DNA, RNase-free RQI treatment was performed according to the manufacturer’s instructions (Promega, Madison, WI, USA), and the samples were further purified using the RNeasy mini kit (Qiagen, Valencia, CA, USA). For RNA-seq analysis, a total of 8 RNA-seq libraries were generated, comprising four biological replicates from each of the two vineyards (V7 and V9). The libraries were constructed as previously described (Bai et al., 2014) using the NEBNext Ultra II RNA Library Prep Kit for Illumina (New England Biolabs, Ipswich, MA). Subsequently, these libraries were pooled in equal amounts and subjected to paired-end 150-base sequencing on two lanes of the NovaSeq 6000 platform (Illumina, San Diego, CA) at the Novogene Co., Ltd (Sacramento, CA).

### 2.5 RNA-seq data preprocessing and Identification of differentially expressed genes

Illumina sequencing of the multiplexed RNA-seq libraries resulted in 8 FASTQ files containing sequences, and the data processing followed the methods described in our previous work (Ismail et al., 2022a). In summary, the quality of reads was assessed using FASTQ (https://www.bioinformatics.babraham.ac.uk/projects/fastqc/) before and after trimming with Trimmomatic v0.39 (**Supplementary Table 1**; Bolger et al., 2014). Subsequently, the trimmed reads were quantified using Salmon in non-alignment-based mode to estimate transcript abundance (Patro et al., 2017). The transcripts were mapped to the Vitis transcriptome file “Vvinifera_457_v2.1.transcript_primaryTranscriptOnly.fa” extracted from Phytozome database (http://www.phytozome.net), resulting in a mapping rate higher than 61.9% (**Supplementary Table 1**). To identify differentially expressed genes (DEGs) between V7 and V9 at the sampling point, we utilized the DESeq2 and EdgeR packages with default parameters (Love et al., 2014; Chen et al., 2016). For convenience, the DEGs generated by both DESeq2 and EdgeR pipelines, with a threshold of PFDR<0.05 and log2fold change > 1.5 or < –1.5, were considered as being expressed (**Supplementary Figure 5**). For the analysis of Gene Ontology (GO) terms, we employed the g:Profiler website with the g:SCS multiple testing correction method, using a significance threshold of 0.05 (Raudvere et al., 2019). Finally, to visualize the consensus result, the Web-based tool Venny was used (https://bioinfogp.cnb.csic.es/tools/venny/index.html).

### 2.6 Weighted gene co-expression network analysis

Co-expression network modules were constructed using the variance stabilizing transformation values (VSD) and the R package WGCNA (v1.69; Langfelder and Horvath, 2008). Before analyzing the data, lowly expressed genes among all sample types were removed by DESeq2, and the remaining non-lowly expressed genes of the 8 samples (n = 17,553) were used in module construction. The co-expression modules were obtained using the default settings, except that the soft threshold power was set to 9, TOMType was set to signed, minModuleSize was set to 30, mergeCutHeight was set to 0.25, and scale-free topology fit index was set to 0.8 (R² = 0.8). A module eigengene (ME) value, which summarizes the expression profile of a given module as the first principal component, was calculated and used to evaluate the association of modules with berry biochemical characteristics (tannins, catechin, Quercetin glycosides, and TAC) of V7-berries and V9-berries at the fifth sampling time (S5). The resultant final WGCNA matrix had 42 modules (M1-M42) with 17,553 genes. The module membership (MM) and gene significance (GS) values were calculated, subsequently the intramodular hub genes were identified (GS > 0.2, MM > 0.8, and p-value < 0.05) (WGCNA; Langfelder and Horvath, 2008).

### 2.7 GO enrichment and KEGG pathway analyses

GO and KEGG enrichment analyses were performed using the g:Profiler website with the Benjamini-Hochberg multiple testing correction method, applying a threshold of PFDR < 0.05 (Raudvere et al., 2019). The non-redundant biological process (BP) terms and KEGG pathways for differentially expressed genes (DEGs) located in modules M21 and M30, as well as the 31 genes of interest, were visualized using the Cytoscape plug-in ClueGO (Bindea et al., 2009).

### 2.8 Validation of DEG subsets by qPCR

cDNA synthesis was carried out using the qScript™ cDNA Synthesis kit (Quanta Biosciences, MD, USA) with total RNA as the template. For quantitative real-time PCR (qRT-PCR), specific primers for the selected genes were designed using the Primer Express 2.0 software (Applied Biosystems) and are listed in **Supplementary Table 8**. The qPCR assays were designed to target the predicted genes, and ubiquitin was used as an internal control gene for normalization. The relative abundance of target mRNA expression levels was determined using the comparative Cq method. The qRT-PCR assays were performed in triplicate, following the MIQE guidelines (Bustin et al., 2009), using the PowerTrackTM SYBR Green Master Mix kit (Applied Biosystems, CA, USA). The reaction mix included 5 µL of 2X master mix, 3.2 µL of nuclease-free water, 0.8 µL of primer mixture (final concentration of 500nM for each forward and reverse primer), and 1 µL of cDNA template. The qPCR was carried out using the QuantStudio 12K Flex Real-Time PCR system (Applied Biosystems, CA, USA) with the following thermal cycling conditions: 50°C for 2 min, 95°C for 2 min, followed by 40 cycles of 95°C for 15 s and 60°C for 1 min. A melting curve analysis was performed from 60 to 95°C with a continuous ramp rate of 1.6°C/s. Relative quantification for each target gene was calculated by the 2–ΔΔT method (Livak and Schmittgen, 2001).

### 2.9 Statistical analyses

The statistical analysis was performed using IBM SPSS Statistics 22 software with a multivariate ANOVA. Results were expressed as mean ± SE of three biological replicates and different letters indicate significant differences between genotypes according to Duncan’s test (P<0.05).

## 3 Results

### 3.1 Berry astringency of Scarlet Royal is positively correlated to its tannins

Scarlet Royal table grape is one of the major red varieties in California. Despite the premium fruit quality of the variety, in some cases, an undesirable taste was observed under certain unknown circumstances. To gain comprehensive insights into the development of the occasional berry astringency of Scarlet Royal and understand the underlying mechanism of this phenomenon, berries were investigated at two contrasting vineyards (V7 and V9), both following the same commercial cultural practices. However, leaf petioles (LP) analysis of grapes from both vineyards (hereafter, LP-V7 and LP-V9) showed considerable differences in nutrient levels, especially in the primary macronutrients (**Supplementary Table 2**). During both seasons, the amount of nitrogen (N) in the form of nitrate (NO_3_^-^) in LP-V9 was roughly 2 to 3 times higher than the normal levels, in contrast to its counterpart in LP-V7, which slightly accumulated more or less N. Similarly, LP-V9 contained higher percentages of phosphorus (P) and potassium (K) compared to LP-V7 (**Supplementary Table 2**). Conversely, the amounts of secondary macronutrients, calcium (Ca) and magnesium (Mg), in LP-V7 were within the normal range but greater than LP-V9, which showed Mg deficiency in the first year only. Regarding the micronutrients, their levels were mainly within or around the normal range at both vineyards and during both seasons, with some differences (**Supplementary Table 2**). For example, zinc (Zn) was slightly higher in LP-V9, especially in the first year. On the contrary, manganese (Mn) and chlorine (Cl) were roughly 2 times higher in V7 (**Supplementary Table 2**). Similarly, soil analysis shoed a higher level of nitrogen, potassium and magnesium (**Supplementary Figure 13**). However, no significant difference was observed in all other soil macro and micronutrients.

During the two seasons of the study, we determined the total marketable yield and the number of clusters in both vineyards. Our data revealed a higher yield in V7 (34.7 ± 1.1 kg and 34.0 ± 3.5 kg) compared to V9 (28.0 ± 1.4 kg and 24.4 ± 4.2 kg) in 2016 and 2017, respectively. The lower yield in V9 can likely be attributed to the smaller number of clusters in V9 (27.7 ± 1.5 and 32.2 ± 0.3) compared to V7 (37.2 ± 5.6 and 45.7 ± 1.7) during 2016 and 2017.

**Figure 1:**
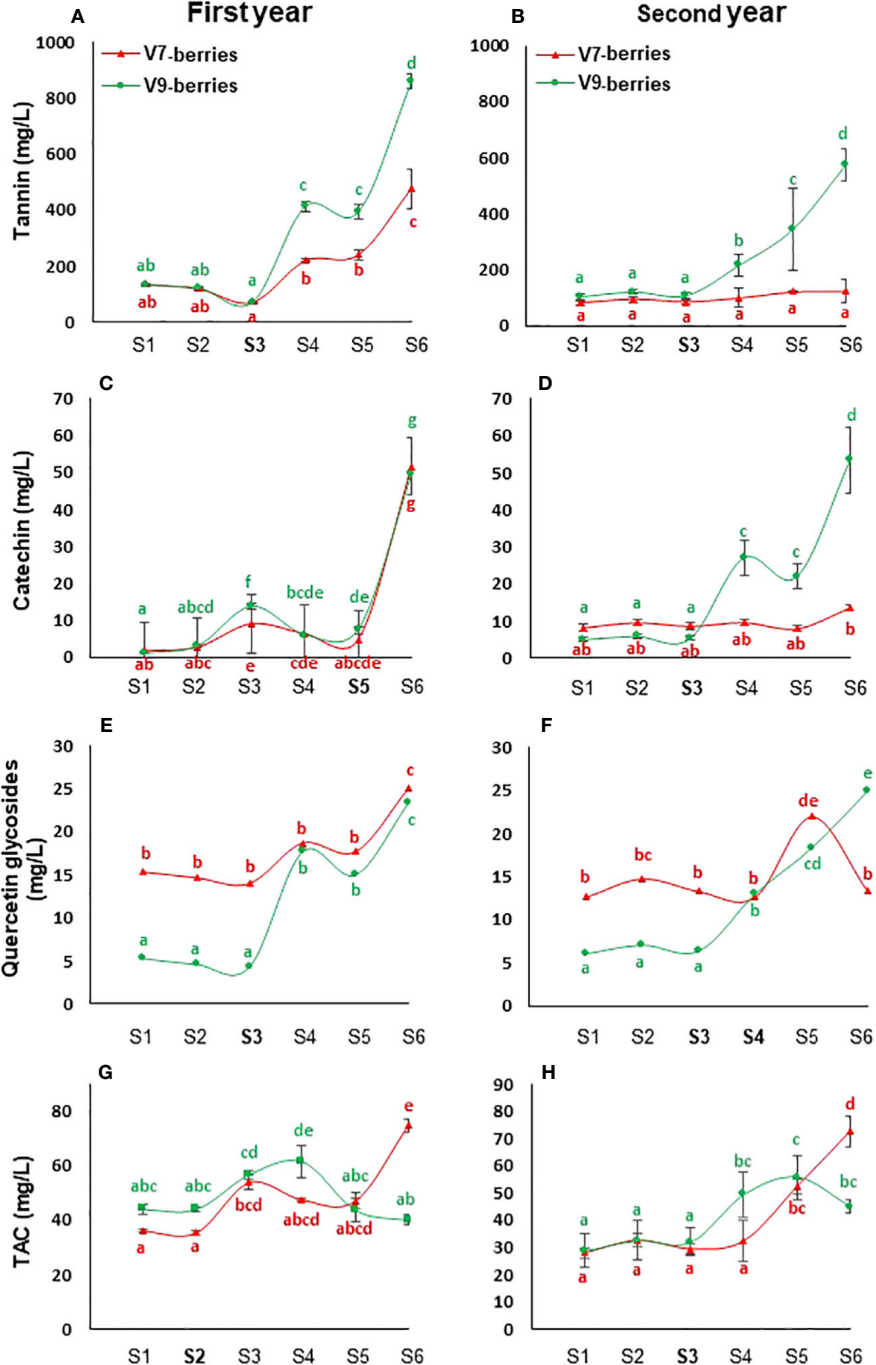
Polyphenols content of Scarlet Royal cultivar in V7-berries and V9-berries. The amounts of tannins **(A, B)**, catechin **(C, D)**, quercetin glycosides **(E, F)**, and total anthocyanins TAC **(G, H)** in the berries of Scarlet Royal grapevine that was cultivated at two California vineyards V7 and V9, exhibiting low and high stringent taste, respectively, during the first **(A, C, E, G)** and second **(B, D, F, H)** year. Error bars represent standard error of the mean (n = 3).

To monitor the changes in the biochemical composition of Scarlet Royal berries, V7 and V9 berries were periodically sampled at six time points from veraison until the end of the season (S1 – S6, see the materials and methods section). The obtained data showed that berry polyphenols exhibited discernible patterns in both vineyards, most notably during the ripening stage (**Figure 1**). Of special interest were the tannin compounds, which widely affect organoleptic properties such as astringency and bitterness (Soares et al., 2020). Our data showed that berries from both V7 and V9 vineyards maintained lower levels of tannin from veraison up to the middle of August (at time point S3, **Figures 1A, B**). Subsequently, a significant gradual increment of tannin took place. However, only V9-berries showed consistent accumulation of tannin over the two studied seasons compared to V7-berries, where the significant induction occurred only during the first season. It is worth noting that the levels of tannin were lower in both vineyards during the second year compared to the first season. Nevertheless, they were more pronounced in V9-berries compared to V7-berries, with roughly 2- to 4.5-fold increases by the end of the harvesting time during the two seasons, respectively (**Figures 1A, B**).

The patterns of catechin and quercetin glycosides were inconsistent during both seasons, particularly within V7-berries (**Figures 1C, F**). During the first year, for instance, the levels of catechin were similar in both vineyards, showing a dramatic increase only by the end of the season (at the time of S6, **Figure 1C**). In contrast, during the second year, such induction of catechin was exclusively restricted to V9-berries, starting from time S3 (**Figure 1D**). For quercetin glycosides, V7-berries exhibited significantly higher amounts at early stages (sampling time S1 – S3) during both seasons relative to V9-berries (**Figures 1E, F**). However, subsequent amounts were comparable in both vineyards during the first season only (sampling time S4 – S6, **Figure 1E**), but not in the second one, where V7-berries showed a significant drop at the last sample S6 (**Figure 1F**). Interestingly, the levels of quercetin glycosides were roughly equal at the last V9-berries sample (S6) between both seasons despite such inconsistency. For total anthocyanins (TAC), the levels in early samples were comparable in both vineyards and seasons (sampling time S1 – S3, **Figures 1G, H**). Afterwards, their pattern started to vary between V7 and V9 within the same season, as well as from the first season to the second, as the nutrient amounts fluctuated as well (**Supplementary Table 2**). Nevertheless, TAC accumulation was positively correlated with the progress of ripening in V7-berries, but not V9-berries.

To further confirm our data, we measured these phenolic compounds for the third time in mid-September (S5) of the next (third) year (**Supplementary Figure 1**). Overall, the results showed that the patterns of tannins and TAC were reciprocally inverted between V7-berries and V9-berries as ripening advanced. In addition, both catechin and quercetin glycosides most likely followed the pattern of tannins despite their seasonal fluctuations.

To further distinguish V7-berries and V9-berries and assess their astringency development, a panel test was performed using samples at three commercial harvest times (S3, S4, and S5). A group of 12 nontechnical panelists scored berry astringency on a scale from 1 to 7, where 1 is extremely low and 7 is extremely high. The panelists were trained using samples from contrasting standard varieties, including Flame Seedless and Crimson as non-astringent and Vintage Red known for its astringent taste (Personal communication).

**Figure 2:**
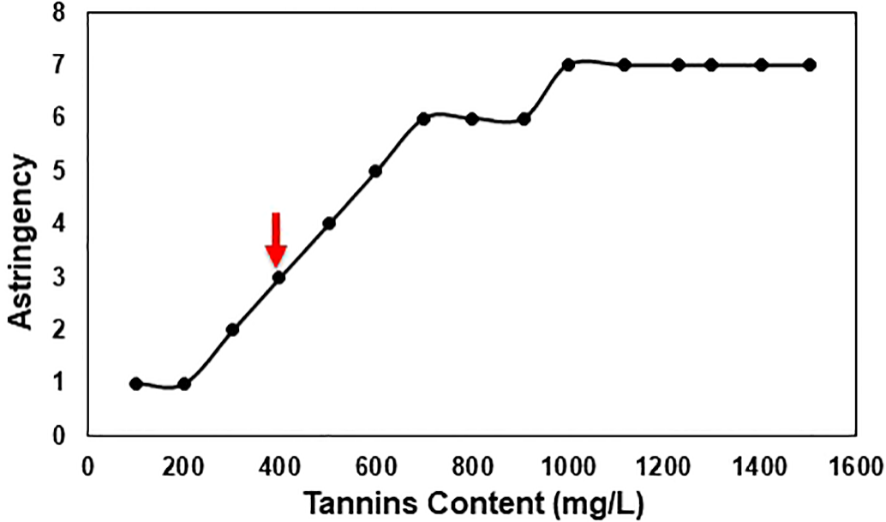
Scarlet Royal astringency in relation to tannins content, an arrow indicates the threshold for the astringent taste. Clusters were sorted based on taste using the 12 panelists and then samples of these clusters were collected and sent to the ETS laboratory for phenolic compound analysis.

The results showed that V7-berries exhibited lower intensity of astringency compared to V9-berries (**Supplementary Figure 2**). As ripening proceeded, astringency levels increased in V9-berries, but decreased in V7-berries. Moreover, we collected samples from clusters with various astringent taste and measured its tannins content. We were able to determine that the threshold level of tannins that causes the Scarlet Royal astringency taste is around 400 mg/L (**Figure 2**).

Taking into account the levels of polyphenol compounds (**Figure 1**) and the taste panel data together (**Supplementary Figure 2**), it is evident that astringency development is positively associated with tannins’ accumulation throughout the ripening process of V9-berries. Nevertheless, organoleptic analysis revealed a significant difference in the berries of the two vineyards, particularly in terms of total soluble solids and titratable acidity (**Supplementary Figure 14**). Notably, V9 berries exhibited higher titratable acidity and lower total soluble solids, especially in the later stages (S3, S4, and S5). It’s worth noting that the weight of V9 berries is also higher than that of V7 (**Supplementary Figure 14**).

### 3.2 Changes in berry transcriptome of astringent and non-astringent Scarlet Royal berries

**Figure 3:**
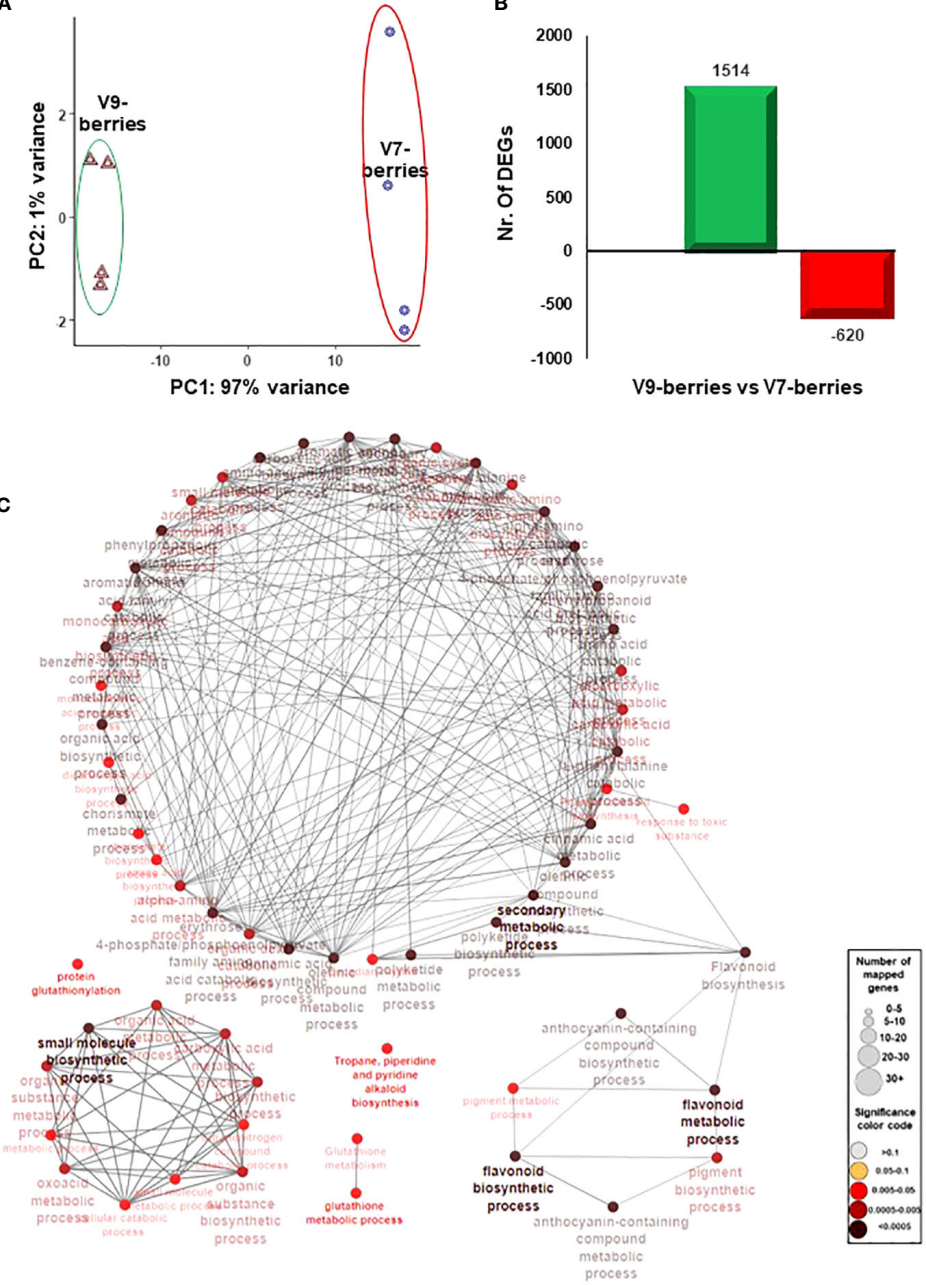
**(A)** The principal components analysis (PCA) of the Scarlet Royal berry samples, where normalized counts were transformed to the variance stabilizing transformation (VST) using DESeq2. The V7-berries and V9-berries were given a distinctive color. **(B)** Bar plot of differentially expressed genes (DEGs) when 9-berries were compared against 7-berries using DESeq2 or EdgeR pipelines resulted in non-redundant 2134 genes. The fold2change of those genes are higher than 1.5 or lower than -1.5 (*p*-adjusted < 0.05). **(C)** The network of the predefined Biological Processes GO terms and KEEG that are overrepresented in the up-regulated DEGs of V9-berries using the g:Profiler website with Benjamini-Hochberg FDR multiple testing correction method. The default ClueGO settings were applied, and the terms are functionally grouped based on shared genes (kappa score). The size of the nodes indicates the number of mapped genes, while the color indicates the groups. The most significant term defines the name of the group.

To better understand the molecular events associated with the induction of tannins and astringency upon ripening, the berry transcriptome profile was analyzed in both V7-berries and V9-berries at the late commercial harvest date (S5). Following the quality and quantity check, extracted RNA from quadruplicate samples was deeply sequenced (GenBank accession number: PRJNA998369). Of the 19.7 to 24.4 million high-quality clean reads per replicate, 61.9% to 66.1% were mapped against the *V. vinifera* transcriptome (**Supplementary Figure 3**, **Supplementary Table S1**). Hierarchical clustering of the RNAseq data showed explicit changes in the berry transcriptome profile between V7-berries and V9-berries (**Supplementary Figure 4**). The Principal Component Analysis (PCA) showed high consistency among biological replicates (**Figure 3A**). Samples were mainly separated along the first component (PC1), which was responsible for 97% of the variance, and was definitely associated with the site of cultivation; V7 and V9. In contrast, the second component (PC2) was trivial, accounting for only 1% of the variance and was probably attributed to experimental error. Such results were expected, as berry samples came from the same cultivar, Scarlet Royal (in other words, same genetic background), and the only difference between them was the vineyard locations.

To identify the differentially expressed genes (DEGs) in V7-berries and V9-berries at this specific time within the ripening window, the RNAseq data were analyzed using two different Bioconductor packages, DESeq2, and EdgeR (Love et al., 2014; Chen et al., 2016). Subsequently, the DEGs with FDR < 0.05 and log2fold change > 1.5 or < –1.5 generated by both pipelines were considered (**Supplementary Figure 5**). The pairwise comparison between berry transcriptomes (V9-berries vs V7-berries) resulted in 2134 DEGs, with 1514 up-regulated and 620 down-regulated (**Figure 3B**). The data manifested the impact of the cultivation site on the transcriptional reprogramming of a large number of genes that ultimately affect berry quality. Most apparently, at the V9 vineyard, where roughly 2.5-fold higher number of berry transcripts were up-regulated compared to V7 (**Figure 3B**, **Supplementary Table 3**).

Subsequently, the enrichment of Gene Ontology (GO) terms and Kyoto Encyclopedia of Genes and Genomes (KEGG) pathways were analyzed among the up- and down-regulated DEGs using the Vitis vinifera Ensembl GeneID (Raudvere et al., 2019; **Supplementary Table 4**). Among the significantly enriched GO terms, the up-regulated transcripts in V9-berries exhibited high enrichment in the molecular function (MF) GO terms for quercetin 3-O-glucosyltransferase activity (GO:0080043) and quercetin 7-O-glucosyltransferase activity (GO:0080044) (**Supplementary Figure 6**, **Supplementary Table 4**). Additionally, the V9-berries induced DEGs were highly enriched in the biological process (BP) GO terms for the jasmonic acid signaling pathway and cellular response (GO:2000022, GO:0009867, GO:0071395, GO:0009753), L-phenylalanine metabolic process (GO:0006558), L-phenylalanine biosynthetic process (GO:0009094), and nitrogen compound metabolic process (GO:0006807). Similarly, these DEGs were highly enriched in the KEGG pathways for the biosynthesis of secondary metabolites (KEGG:01110) and phenylpropanoid biosynthesis (KEGG:00940) (**Figure 3C**, **Supplementary Table 4**). On the other hand, the down-regulated transcripts in V9-berries (but induced in V7-berries) showed substantial augmentation in the MF GO terms for hormone binding (GO:0042562), abscisic acid binding (GO:0010427), and potassium ion transmembrane transporter activity (GO:0015079) (**Supplementary Figure 7**, **Supplementary Table 4**). Correspondingly, the BP GO terms for hormone-mediated signaling pathway and response (GO:0009755 and GO:0009725), auxin-activated signaling, cellular response, and homeostasis (GO:0009734, GO:0071365, GO:0010252), abscisic acid-activated signaling, response, and cellular response (GO:0009738, GO:0009737, GO:0071215), response to strigolactone (GO:1902347), potassium ion transmembrane transport (GO:0071805), and potassium ion transport (GO:0006813), as well as the KEGG pathways for plant hormone signal transduction (KEGG:04075), brassinosteroid biosynthesis (KEGG:00905), and carotenoid biosynthesis (KEGG:00906) were highly enriched in the down-regulated genes of V9-berries (**Supplementary Figure 8**, **Supplementary Table 4**). Overall, the transcriptome analysis pointed out the substantial changes in transcript abundance that coordinate and reflect the observed induction of tannins/astringency during the maturation and ripening of V9-berries compared to the V7-berries (**Figures 1**, **2**, **Supplementary Figure 2**).

### 3.3 Identifying co-expressed genes associated with tannins accumulation

**Figure 4:**
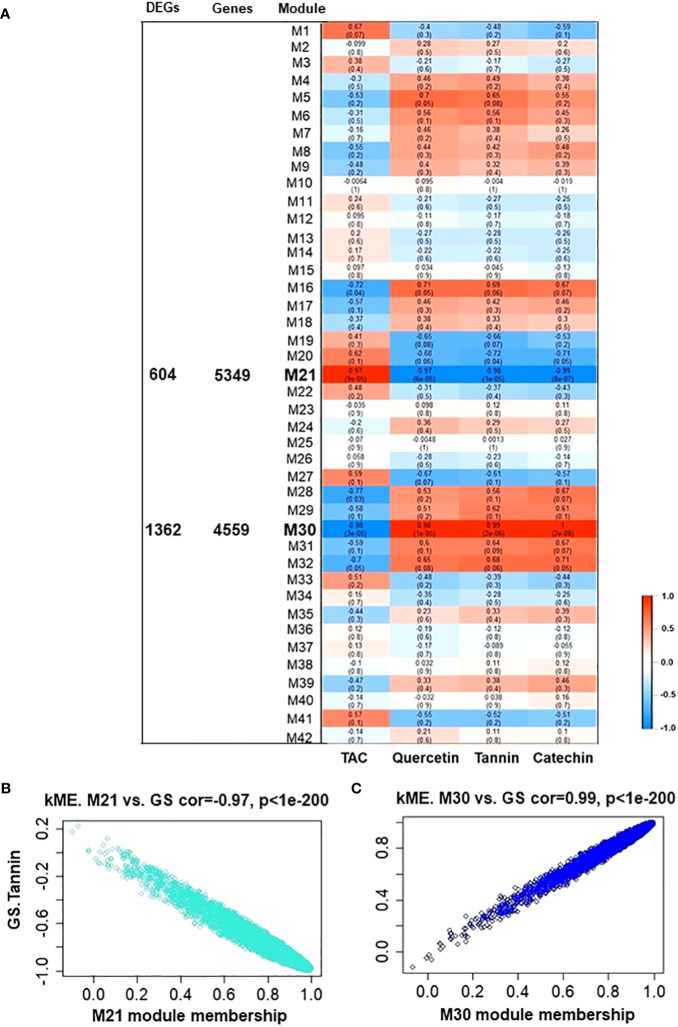
Module-trait associations and gene relationship to trait in WGCNA 21 and 30 module. **(A)** Module-trait correlation between RNA-seq data and polyphenolic-related traits, including tannins, catechin, quercetin glycosides, and total anthocyanin (TAC) from both V7-berries and V9-berries at the late commercial harvest date (S5). The correlation coefficient between a given module and biochemical-related traits is indicated by the color of the cell at the row-column intersection. Each row corresponds to a module (M1-M42). The left panel shows the assigned number of the total input genes or DEGs in each module of interest, M21 and M30 (bold-line) that were selected for further analysis. Blue and red are the color keys that represent *r^2^* values from -1 to 1. **(B, C)** showed gene significance of module M21 and M30, respectively. GS.Tannin is the gene significance for total tannin. Module eigengene connectivity (kME) was calculated for each gene within the M21 and M30 module.

To elucidate which fundamental processes were altered during tannins/astringency induction within berries, the Weighted Gene Co-Expression Network Analysis (WGCNA) was applied to construct co-expression networks. Forty-two modules were identified based on pairwise correlations among the 17553 non-lowly expressed genes (**Supplementary Figures 9**, **10**). Subsequently, the biochemical data (tannin, catechin, quercetin glycosides, and TAC) from both V7-berries and V9-berries were correlated to the WGCNA modules, and only 2 modules, M21 and M30, displayed substantial correlations with berry polyphenols, containing 5349 and 4559 genes, respectively (**Figure 4**). The M21 module was positively linked with TAC (*r^2^* ≥ 0.97), but negatively associated with tannins, catechin, and quercetin glycosides (*r^2^* = – 0.98, – 0.99, and – 0.97, respectively) (**Figures 4A, B**). On the contrary, the M30 module exhibited a positive correlation with tannins, catechin, and quercetin glycosides (*r^2 =^* 0.99, 1.00, and 0.98, respectively), but was negatively linked with TAC (*r^2^* ≥ – 0.98) (**Figures 4A, C**). The DEGs obtained from the two pipelines were assigned to both M21 and M30, yielding 604 and 1362 genes, respectively (**Figure 4A**). Interestingly, the number of DEGs in each module, M21 and M30, was roughly equal to the down- and up-regulated genes, respectively (**Supplementary Table 3**). To identify flavonoids/tannins-related genes that might result in such astringency diversity between V7-berries and V9-berries, hub genes were searched in the DEGs list of both modules (**Supplementary Table 5**). Only 8 hub genes were identified based on their transcript abundances in V9-berries and predicted functions. However, based on our previous work (Ismail et al., 2022a), we found another 11 genes that are significantly expressed but with a log2FoldChange less than 1.5, and they were included in our further analysis (**Supplementary Table 6**). The enrichment analysis of GO showed considerable enrichment in the BP GO terms for secondary metabolite biosynthetic process (GO:0044550 and GO:0019748), flavonoid biosynthetic/metabolic process (GO:0009813 and GO:0009812), L-phenylalanine metabolic/catabolic process (GO:0006558 and GO:0006559), phenylpropanoid metabolic process (GO:0009698), phenylpropanoid biosynthetic process (GO:0009699), chorismate biosynthetic/metabolic process (GO:0009423 and GO:0046417), cinnamic acid biosynthetic/metabolic process (GO:0009800 and GO:0009803), anthocyanin-containing compound biosynthetic/metabolic process (GO:0009718 and GO:0046283). The KEGG pathway analysis confirmed the BP GO terms, exhibiting enrichment for the biosynthesis of secondary metabolites (KEGG:01110), phenylpropanoid biosynthesis (KEGG:00940), flavonoid biosynthesis (KEGG:00941), and glutathione metabolism (KEGG:00480) (**Supplementary Figure 11**; **Supplementary Table 7**).

### 3.4 Validation of the flavonoids/PAs-related genes in berry

**Figure 5:**
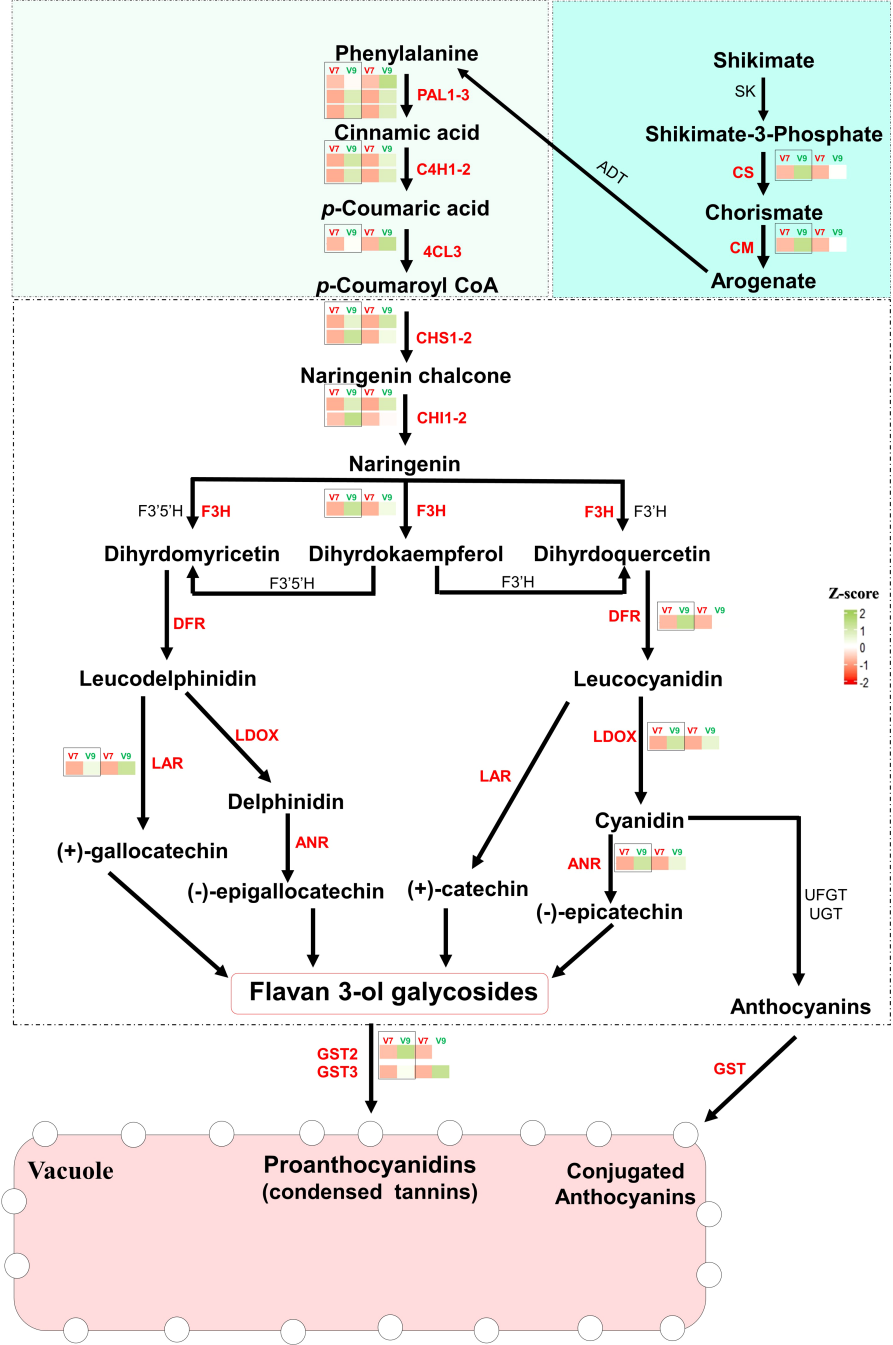
The Flavonoids/PAs biosynthesis pathway. Red-colored genes showed studied genes that are significantly expressed in V9-berries compared to V7-berries, and further studied qPCR. The bar graphs show the induction levels of each gene in each designated pathway, that were studied by qPCR (black box) and RNAseq (indicated as a relative transcript per milion TPM). shikimate dehydrogenase; SK, shikimate kinase 3; CS, Chorismate synthase; CM, chorismate mutase; ADT, arogenate dehydratase/prephenate dehydratase; PAL1, Phenylalanine ammonia-lyase 1; C4H, trans-4-coumarate biosynthesis; 4CL, 4-coumaroyl:CoA-ligase 2; CHS, chalcone synthase; CHI, chalcone isomerase; F3H, flavanone-3-hydroxylase; F3’H, flavonoid 3’-hydroxylase; F3’5’H, flavonoid 3’,5’-hydroxylase; DFR, dihydroflavonol 4-reductase; LODX, leucoanthocyanidin dioxygenase; LAR, leucoanthocyanidin reductase; ANR, anthocyanidin reductase; GST, glutathione S-transferase. The Turquoise box represents the Shikimate pathway. The Light-green box represents the phenylpropanoids pathway. The white box represents the Flavonoids/PAs pathway. The TPM and qPCR values represent the mean of three biological replicates (n = 3).

To precisely elucidate their significance in the tannins/astringency diversity between V7-berries and V9-berries, we studied the expression levels of the 19 hub genes associated with the shikimic and flavonoids pathway. Except for the *PAL1_1* gene (*r^2^* > 0.813, *P* < 0.013), the analysis of their relative expression by real-time quantitative PCR (qPCR) showed a significant correlation (*r^2^* > 0.92, *P* < 0.001) with the Transcripts Per Million (TPM) values for genes of interest, validating the transcriptomic data from both V7- and V9-berries ((**Figure 5**, **Supplementary Figure 12**, **Supplementary Table 8**). In general, all genes showed higher expression levels in V9-berries compared to V7-berries, but with different degrees of induction. For instance, the two genes involved in the shikimic acid pathway, chorismate synthase (*CS*), and chorismate mutase (*CM*), showed visibly higher accumulation abundance in V9-berries at the third harvesting time with approximately 6-fold and 3-fold increases, respectively, compared to V7-berries. Similarly, the upstream structural genes in the phenylpropanoids pathway, including phenylalanine ammonia lyase (*PAL1*, three versions), trans-4-coumarate biosynthesis (*C4H*, two versions), and 4-coumaroyl:CoA-ligase 2 (*4CL*), were significantly induced by approximately 2- to 9-fold in V9-berries. Regarding flavonoids/PAs biosynthesis, chalcone synthase (*CHS*) is considered a key enzyme in this pathway, converting p-coumaroyl-CoA to naringenin chalcone, which is later turned into naringenin by chalcone isomerase (CHI; **Figure 5**). Both genes (*CHS*, two versions, and *CHI*, two versions) were highly expressed (approximately 3- to 13.5-fold) in V9-berries (**Supplementary Figure 12**, **Supplementary Table 8**). Naringenin is subsequently converted by flavonoid 3’-monooxygenase (F3H) to dihydromyricetin and dihydroquercetin, which are further transformed by dihydroflavonol 4-reductase (DFR) into leucodelphinidin and leucocyanidin, respectively (**Figure 5**). The expression levels of F3H and DFR also showed a commensurate induction (approximately 3.6- to 11.2-fold) with the upstream genes in V9-berries relative to V7-berries. Subsequently, leucoanthocyanidin dioxygenase (LDOX) and leucoanthocyanidin reductase (LAR) catalyse the conversion of leucodelphinidin to delphinidin and (+)-gallocatechin, respectively, as well as leucocyanidin to cyanidin and catechin, respectively. These three genes (*LDOX*, *LAR*, and *ANR*) also exhibited a significant increase (approximately 3- to 5.5-fold) in V9-berries. Finally, the expression of genes encoding glutathione S-transferases (GSTs, two versions), one of the most essential anthocyanin transporters, was significantly higher in V9-berries compared to V7-berries, with approximately 3- to 9.2-fold changes (**Supplementary Figure 12**, **Supplementary Table 8**). Our data showed that the expression of flavonoids/PAs related-genes was highly increased in V9-berries at the third harvest time compared to V7-berries, resulting in the accumulation of more PAs in V9-berries.

## 4 Discussion

### 4.1 Tannins accumulation plays a crucial role in astringency development in Scarlet Royal grape

Developing and producing table grapes with high quality is of utmost importance for the success of grapevine breeding programs. Scarlet Royal table grape (*Vitis vinifera L.*), variety (US Plant Patent 16229 P2), is one such success story, producing premium fruit quality and becoming one of the major red varieties in California. However, under certain unknown circumstances, the berry quality of Scarlet Royal grapes can be affected by undesirable astringent taste, which can negatively impact marketability and consumer acceptance. Research on the relationship between astringency and phenolic composition in table grapes is still scarce, especially on the transcriptomic level. In this study, we aimed to understand the molecular events involved in the development of berry astringency, which is a complex set of sensations resulting from the shrinking, drying, drawing, or puckering of the mouth epithelium (ASTM, 2004). We focused on Scarlet Royal berries from two different vineyards (V7 and V9) with contrasting astringency and analyzed the changes in phenolic-related compounds at six different time points from veraison until the last harvesting time. Our panel test revealed that the V9-berries were perceived as more astringent, a characteristic that could be attributed to their elevated levels of tannins (**Figure 1**, **Supplementary Figure 2**).

Differences observed between the two vineyards under study indicate that V7 vines yield more compared to V9. This difference may be attributed to the lower cluster count in V9, a factor known to potentially contribute to astringency, as suggested by Cañón et al., 2014 in wine grapes. Additionally, petiole analysis revealed higher levels of nitrogen and potassium in V9 vines compared to V7. Vine nutrient levels contribute to the final berry quality at harvest (Verdenal et al., 2021). These factors may also contribute to the higher levels of tannins detected in V9 berries; however, further research is needed to confirm this theory. It’s worth noting that weather conditions can play a role in inducing astringency. Nevertheless, the two vineyards are located in close proximity to each other, and weather data collected from the same station in the Delano area indicates similar conditions. Therefore, it is unlikely that astringency or higher phenolic compounds are induced by weather factors.

In fact, several studies, mainly in wine, have pointed to PAs as a determining factor for astringency intensity (Brossaud et al., 2001; Lesschaeve and Noble, 2005). For example, Vidal et al. (2018) reported that the total amount of tannins is the most plausible factor for sensory astringency, with flavan-3-ols dimers, trimers, and non-galloylated tetramers contributing to the astringency sensation. The PAs are a group of oligomers and polymers of flavan-3-ols and are the naturally occurring and predominant type of tannins in grapes and wine (Garrido and Borges, 2013). Another study on aronia berry juice confirmed PAs as the key astringent compounds using sensory evaluation and phenolic profile approaches along with *in-vitro* models (saliva precipitation index and mucin turbidity). The study found that PAs with higher degrees of polymerization were responsible for the strong astringent mouthfeel (Huang et al., 2022). The composition of phenolic substances, especially PAs, seems to play a crucial role in determining berry astringency, and further exploration of this relationship is warranted in fresh fruits of different species and cultivars. Understanding the molecular basis of astringency development in Scarlet Royal berries can provide valuable insights for improving grape breeding programs and enhancing the overall quality of table grapes.

### 4.2 Tannins/astringency development is affected by multiple factors

The relationship between astringency and the berry polyphenols content has not been explored yet in table grape at the molecular level. To the best of our knowledge, the present study provides the first transcriptome profiling along with the changes of polyphenols in grape berries of the same variety but having astringency diversity (V7-berries and V9-berries). The transcriptome profile of both V7-berries and V9-berries underlined the remarkable transcriptional shift during berry ripening at different vineyards (**Figure 3B**). Commonly, berry transcriptome profiles may widely vary based on many factors, including genotypic variations among varieties/species and developmental stages (Fasoli et al., 2012; Massonnet et al., 2017; Ismail et al., 2022a; Ismail et al., 2022b), as well as environmental circumstances. In our case of study, stemmed differences from the developmental stages and genotypic variations were eliminated, and hence the difference of the vineyard locations was the main source of variance with 97% of variance (**Figures 3A, B**, **Supplementary Table 3**). The identified DEGs output of V9-berries compared to V7-berries is highly explanatory, including polyphenolic-related genes that are robustly expressed and co-regulated with astringency development, particularly in the V9-berries. The enrichment of the up-regulated genes (the V9-induced) with BP GO terms related to the biosynthesis of secondary metabolites, phenylpropanoid, and nitrogen compound metabolic process (**Figure 3C**, **Supplementary Table 4**), commensurate with the higher amounts of N found in V9-berries (**Supplementary Table 1**). In fact, not only the levels of N fertilization but also its different forms highly affected the composition of phenolic compounds in leaves and wine (Lang et al., 2019). However, the synergistic/antagonistic effects of other macro- and micro-nutrients should also be considered. Our results highlighted the negative impact of above-normal amounts of macronutrients, mainly N, and to a lesser extent P and K, on the desirable attributes of grape berries. Otherwise, the positive effect of Ca, Mg, and Mn were achieved as their levels were maintained within the normal range (**Supplementary Table 1**). These data should be also seen in the light of the highly enriched BP GO terms in the down-regulated genes (the V7-induced). Particularly, those for hormonal signaling pathways such as auxin, abscisic acid, strigolactones, as well as the KEGG pathway for the carotenoid biosynthetic pathway (**Supplementary Figure 8**, **Supplementary Table 4**).

### 4.3 Expression of genes related to flavonoids/PAs biosynthesis in Scarlet Royal berries

The transcriptome profiling identified the common and unique molecular events featuring the development of tannins/astringency in grape berries. It is well-documented that the synthesis of PAs in grapevines is achieved via three sequential pathways: the shikimate pathway, the phenylpropanoid pathway, and ultimately the flavonoid pathway (Wei et al., 2021). Our results revealed that the expression levels of flavonoids/PAs-related genes were highly induced in V9-berries at the third harvesting time compared to V7-berries. The 19 selected genes were involved in the three pathways: the shikimate pathway, phenylpropanoids pathway, and flavonoids pathway. The shikimate pathway is an alternative route to produce aromatic compounds, including phenylalanine, tyrosine, and tryptophan, which serve as precursors for various metabolites, such as phenolic compounds (Shu, 2007; Teixeira et al., 2022). The up-regulation of genes like chorismate synthase (*CS*) and chorismate mutase (*CM*) in V9-berries may lead to the accumulation of phenylalanine, which is a critical precursor for the phenylpropanoid pathway. The latter pathway is responsible for synthesizing several end products, including PAs, anthocyanins, lignin, lignans, hydroxycinnamic acid esters, and hydroxycinnamic acid amides (Gray et al., 2012). Under the conditions of the V9 vineyard, several PAs/flavonoids structural genes such as *PAL*, *C4H*, *4CL*, *CHS*, *CHI*, *F3H*, *LDOX*, *LAR*, and *ANR* were induced in V9-berries, leading to the accumulation of PAs in the berries (**Figure 5**). This process is facilitated by GSTs and transported by multidrug and toxic compound extrusion (MATE) transporters. The activation of the PAs biosynthetic pathway in V9-berries may lead to a reduction in the necessary substrates for anthocyanin synthesis, resulting in low red color intensity in V9-berries compared to V7-berries. Additionally, the accumulation of PAs is associated with the development of astringency taste in V9-berries.

Our study provides valuable insights into the molecular events underlying astringency development in Scarlet Royal berries. By integrating transcriptome profiling with polyphenolic composition analysis, the research shed light on the co-regulation of genes involved in the shikimate, phenylpropanoid, and flavonoid pathways, leading to the synthesis of PAs and ultimately influencing astringency. The findings from this research have implications for grapevine breeding programs and the production of high-quality table grapes. Understanding the molecular mechanisms underlying astringency development can help breeders in selecting and developing grape varieties with desirable attributes. Additionally, the knowledge gained from this study can inform vineyard management practices, such as nutrient fertilization, to optimize polyphenolic composition and berry quality.

In conclusion, the data presented in this study indicates that berry astringency is strongly correlated with a high tannin content, likely resulting from the activation of nineteen genes within the phenylpropanoid pathway. The activation of these genes shifts the flavonoid biosynthesis pathway towards proanthocyanins, leading to increased tannin accumulation in the berries. It appears that triggering these events is associated with nutritional imbalances and a lower number of clusters per vine, as confirmed by petiole nutrient levels and the observed lower berry soluble solids and higher titratable acidity levels. The identification of these genes holds significant value for table grape genetic improvement programs. The nutrient imbalance theory derived from this research could be applied worldwide to optimize grapevine fertilization programs. Furthermore, it paves the way for further research in this area, with a particular focus on vine nutrients, crop load management, and berry astringency, thereby contributing to advancements in the field of table grape cultivation.

## Data availability statement

The datasets presented in this study can be found in online repositories. The names of the repository/repositories and accession number(s) can be found in the article/**Supplementary Material**.

## Author contributions

AI: Conceptualization, Formal Analysis, Investigation, Methodology, Software, Validation, Visualization, Writing – original draft, Writing – review & editing. TP: Formal Analysis, Investigation, Methodology, Writing – review & editing. SC: Formal Analysis, Methodology, Writing – review & editing. SB: Formal Analysis, Methodology, Writing – review & editing. AR: Formal Analysis, Methodology, Writing – review & editing. GV: Formal Analysis, Methodology, Writing – review & editing. IE: Formal Analysis, Methodology, Writing – review & editing. DO: Methodology, Resources, Writing – review & editing. AE: Conceptualization, Data curation, Funding acquisition, Investigation, Methodology, Project administration, Resources, Supervision, Visualization, Writing – review & editing, Writing – original draft.
