# Supplementary material for: Unraveling the occasional occurrence of berry astringency in table grape cv. Scarlet Royal: a physiological and transcriptomic analysis
Source: Front Plant Sci. 2023 Oct 26;14:1271251. doi: 10.3389/fpls.2023.1271251 (PMC10641383; doi:10.3389/fpls.2023.1271251)
Supplement: Supplementary file 11 [file Presentation_1.pptx]

## Slide 1
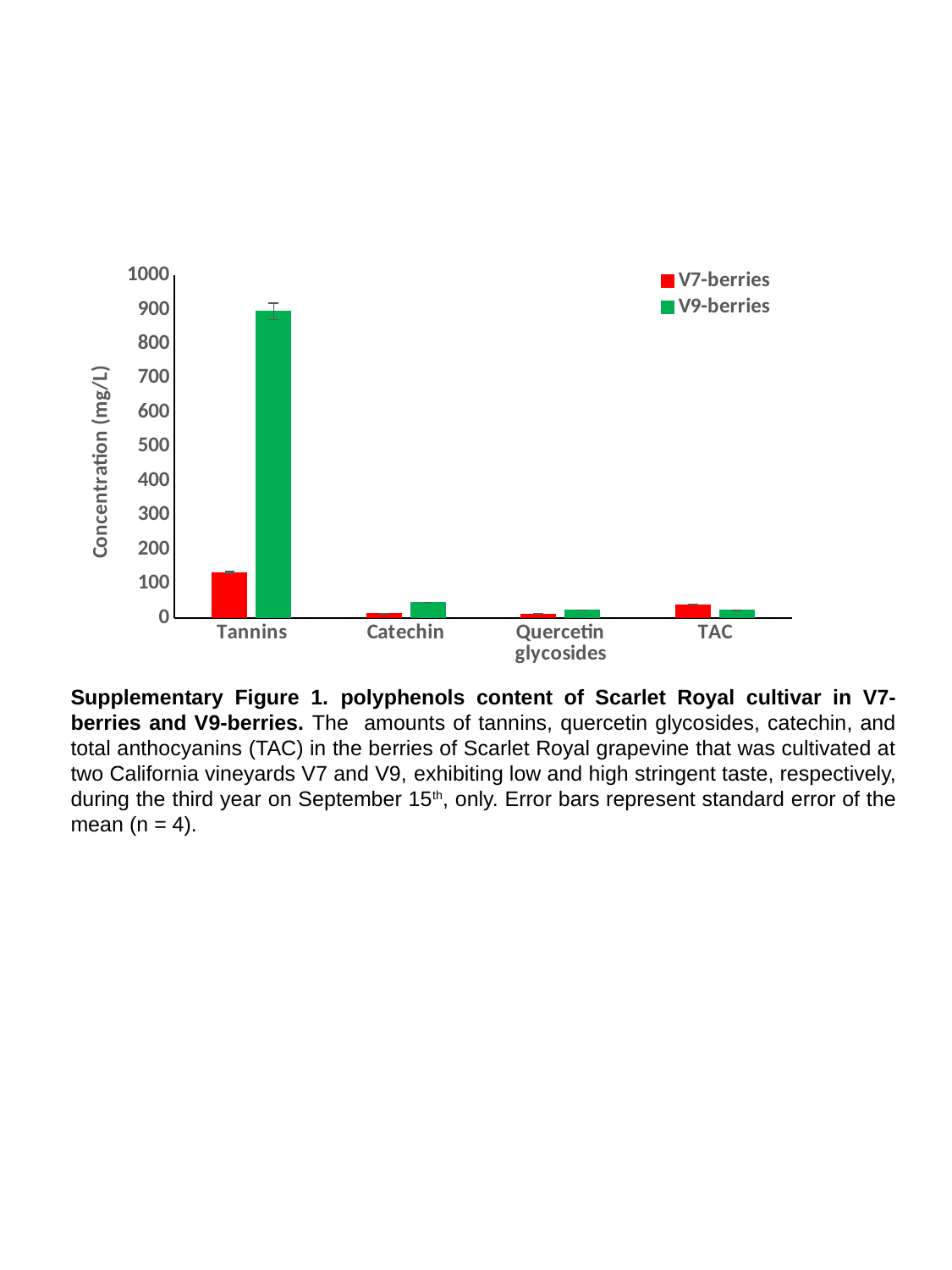

### Chart
| Category | V7-berries | V9-berries |
|---|---|---|
| Tannins | 132.75 | 895.0 |
| Catechin | 12.5 | 45.25 |
| Quercetin glycosides | 11.75 | 23.25 |
| TAC | 38.75 | 22.25 |Supplementary Figure 1. polyphenols content of Scarlet Royal cultivar in V7-berries and V9-berries. The amounts of tannins, quercetin glycosides, catechin, and total anthocyanins (TAC) in the berries of Scarlet Royal grapevine that was cultivated at two California vineyards V7 and V9, exhibiting low and high stringent taste, respectively, during the third year on September 15th, only. Error bars represent standard error of the mean (n = 4).

## Slide 2
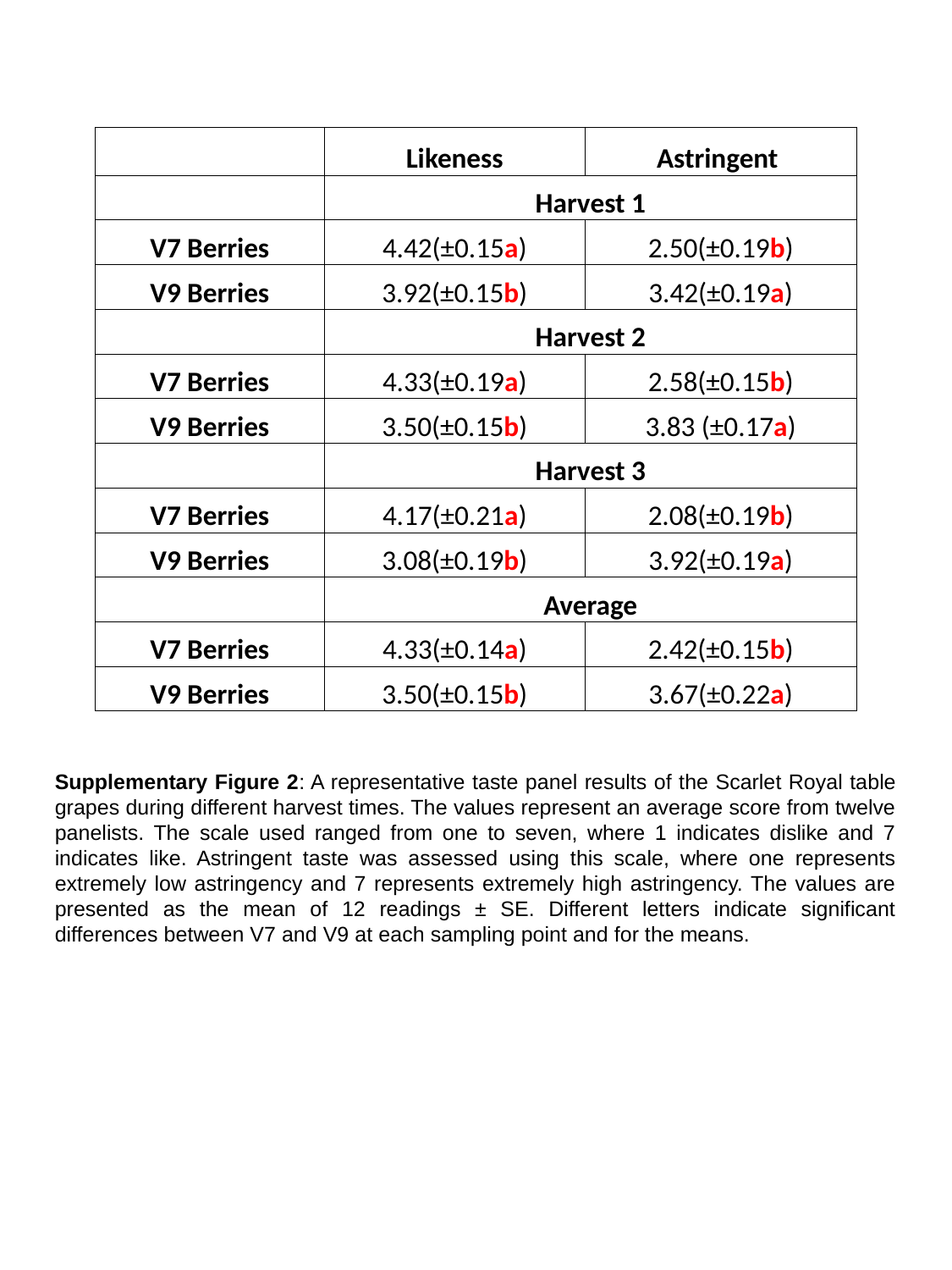

| | Likeness | Astringent |
| --- | --- | --- |
| | Harvest 1 | |
| V7 Berries | 4.42(±0.15a) | 2.50(±0.19b) |
| V9 Berries | 3.92(±0.15b) | 3.42(±0.19a) |
| | Harvest 2 | |
| V7 Berries | 4.33(±0.19a) | 2.58(±0.15b) |
| V9 Berries | 3.50(±0.15b) | 3.83 (±0.17a) |
| | Harvest 3 | |
| V7 Berries | 4.17(±0.21a) | 2.08(±0.19b) |
| V9 Berries | 3.08(±0.19b) | 3.92(±0.19a) |
| | Average | |
| V7 Berries | 4.33(±0.14a) | 2.42(±0.15b) |
| V9 Berries | 3.50(±0.15b) | 3.67(±0.22a) |
Supplementary Figure 2: A representative taste panel results of the Scarlet Royal table grapes during different harvest times. The values represent an average score from twelve panelists. The scale used ranged from one to seven, where 1 indicates dislike and 7 indicates like. Astringent taste was assessed using this scale, where one represents extremely low astringency and 7 represents extremely high astringency. The values are presented as the mean of 12 readings ± SE. Different letters indicate significant differences between V7 and V9 at each sampling point and for the means.

## Slide 3
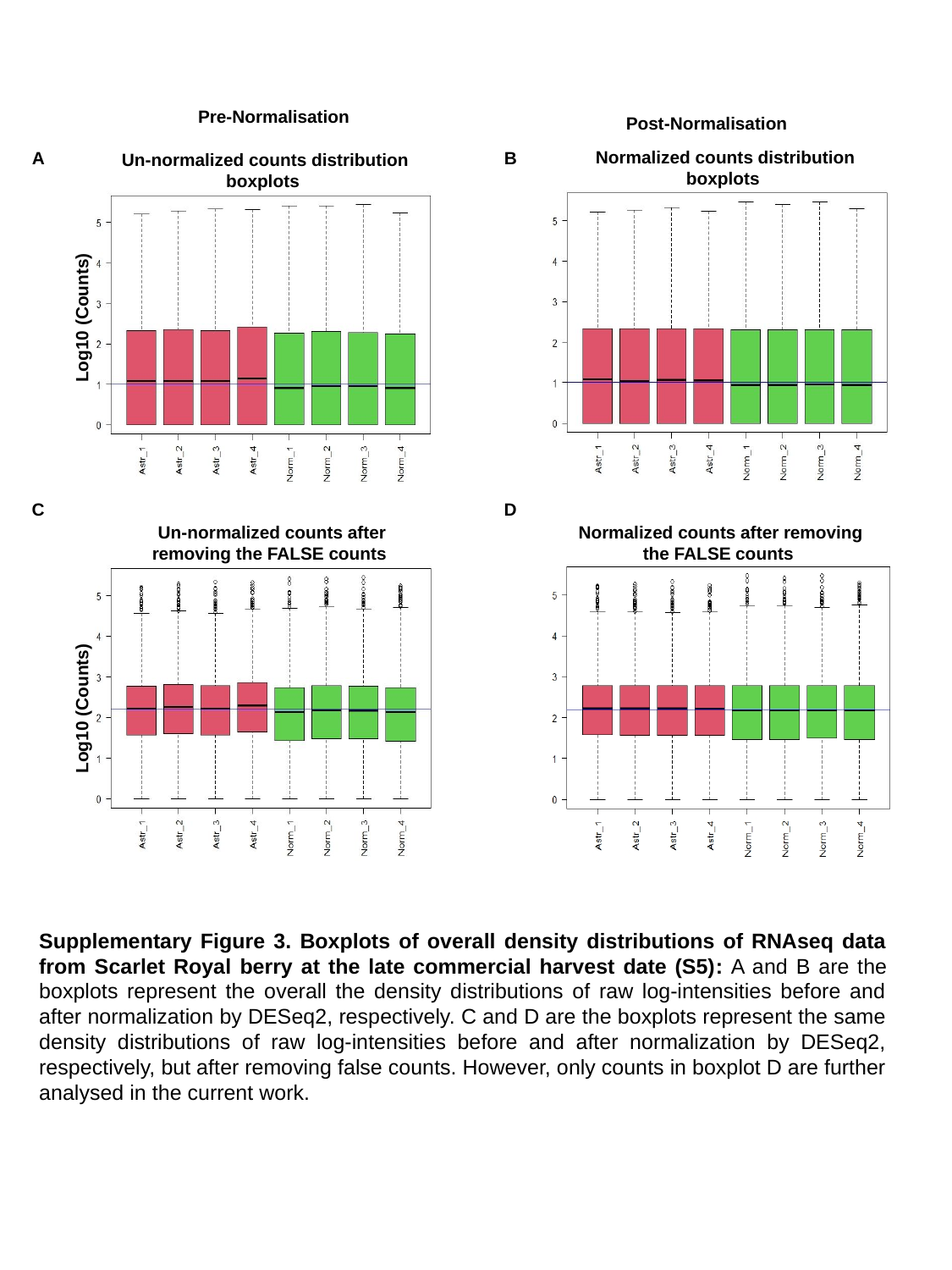

Pre-Normalisation
Post-Normalisation
 Normalized counts distribution boxplots
A
B
 Un-normalized counts distribution boxplots
Log10 (Counts)
C
D
Un-normalized counts after removing the FALSE counts
Normalized counts after removing the FALSE counts
Log10 (Counts)
Supplementary Figure 3. Boxplots of overall density distributions of RNAseq data from Scarlet Royal berry at the late commercial harvest date (S5): A and B are the boxplots represent the overall the density distributions of raw log-intensities before and after normalization by DESeq2, respectively. C and D are the boxplots represent the same density distributions of raw log-intensities before and after normalization by DESeq2, respectively, but after removing false counts. However, only counts in boxplot D are further analysed in the current work.

## Slide 4
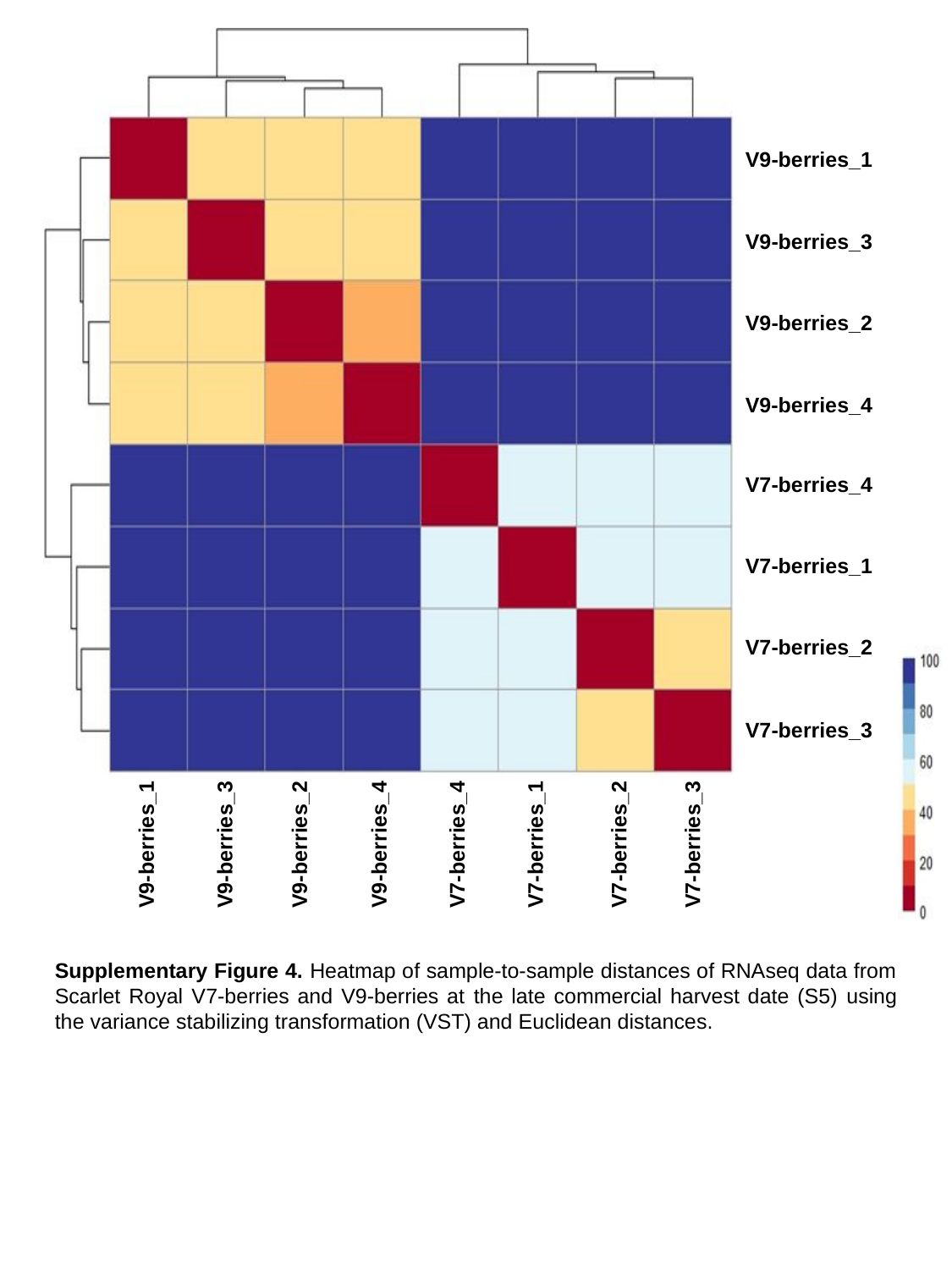

V9-berries_1
V9-berries_3
V9-berries_2
V9-berries_4
V7-berries_4
V7-berries_1
V7-berries_2
V7-berries_3
V9-berries_3
V9-berries_2
V9-berries_4
V7-berries_4
V7-berries_1
V7-berries_2
V7-berries_3
V9-berries_1
Supplementary Figure 4. Heatmap of sample-to-sample distances of RNAseq data from Scarlet Royal V7-berries and V9-berries at the late commercial harvest date (S5) using the variance stabilizing transformation (VST) and Euclidean distances.

## Slide 5
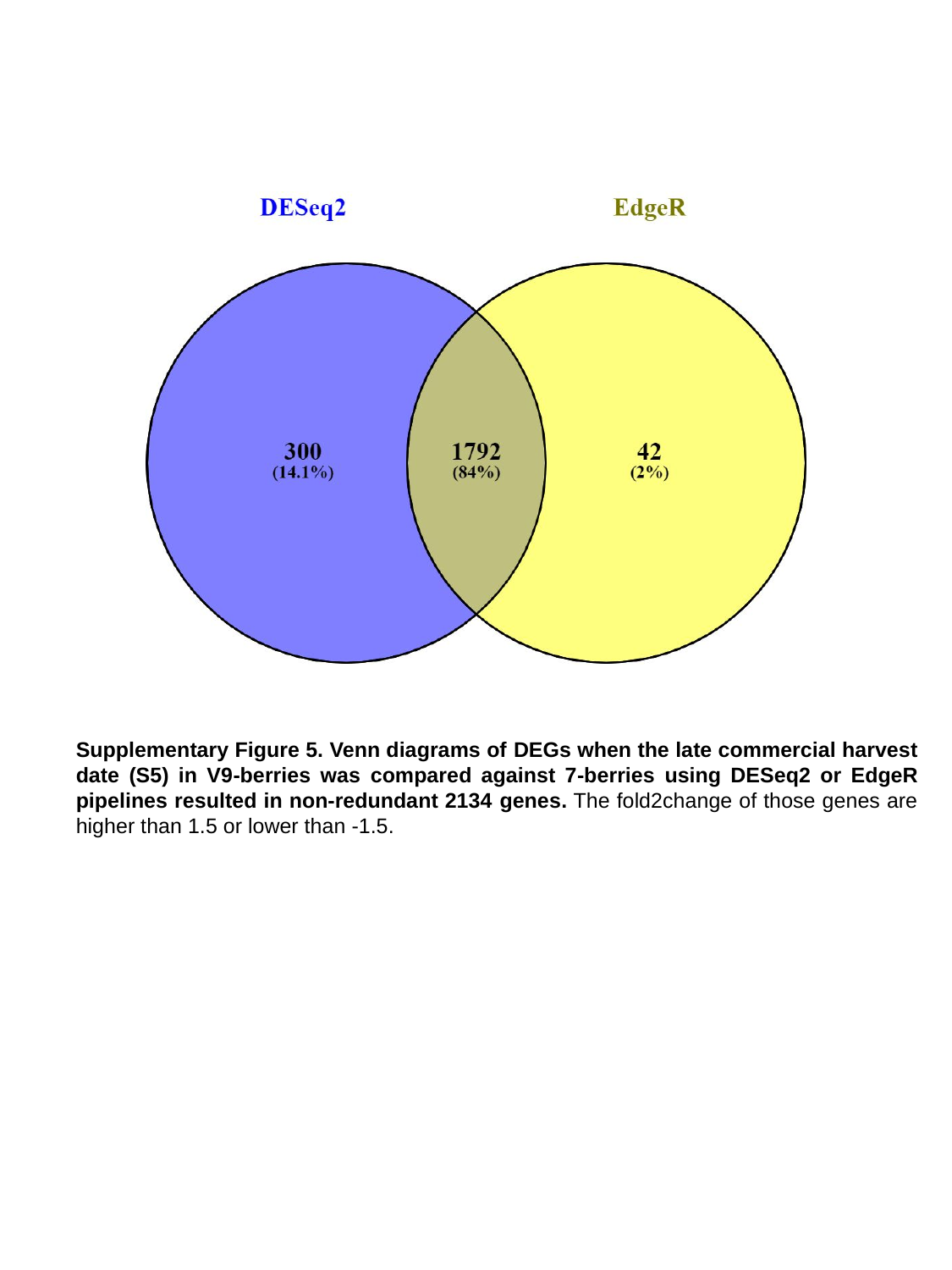

Supplementary Figure 5. Venn diagrams of DEGs when the late commercial harvest date (S5) in V9-berries was compared against 7-berries using DESeq2 or EdgeR pipelines resulted in non-redundant 2134 genes. The fold2change of those genes are higher than 1.5 or lower than -1.5.

## Slide 6
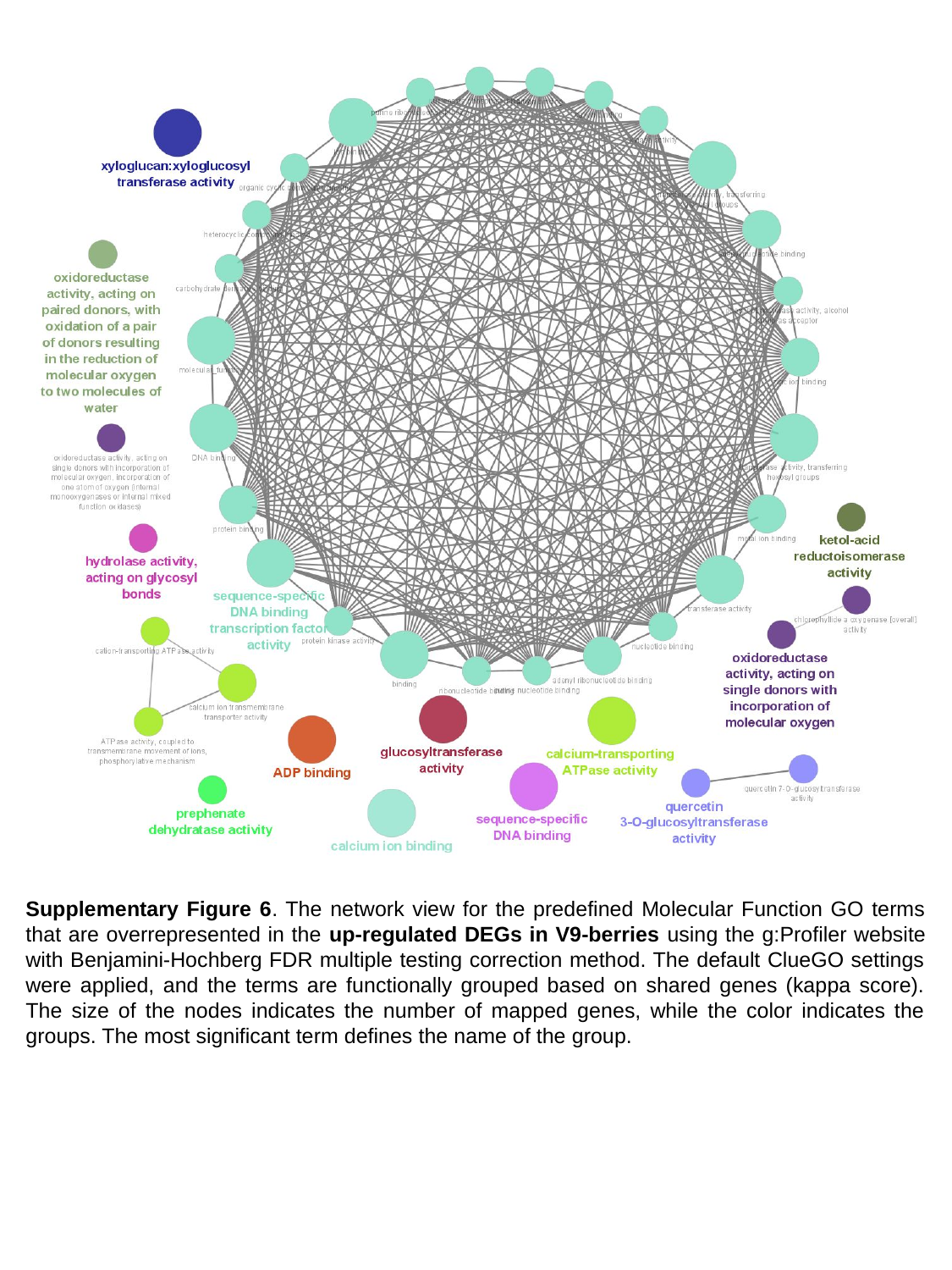

Supplementary Figure 6. The network view for the predefined Molecular Function GO terms that are overrepresented in the up-regulated DEGs in V9-berries using the g:Profiler website with Benjamini-Hochberg FDR multiple testing correction method. The default ClueGO settings were applied, and the terms are functionally grouped based on shared genes (kappa score). The size of the nodes indicates the number of mapped genes, while the color indicates the groups. The most significant term defines the name of the group.

## Slide 7
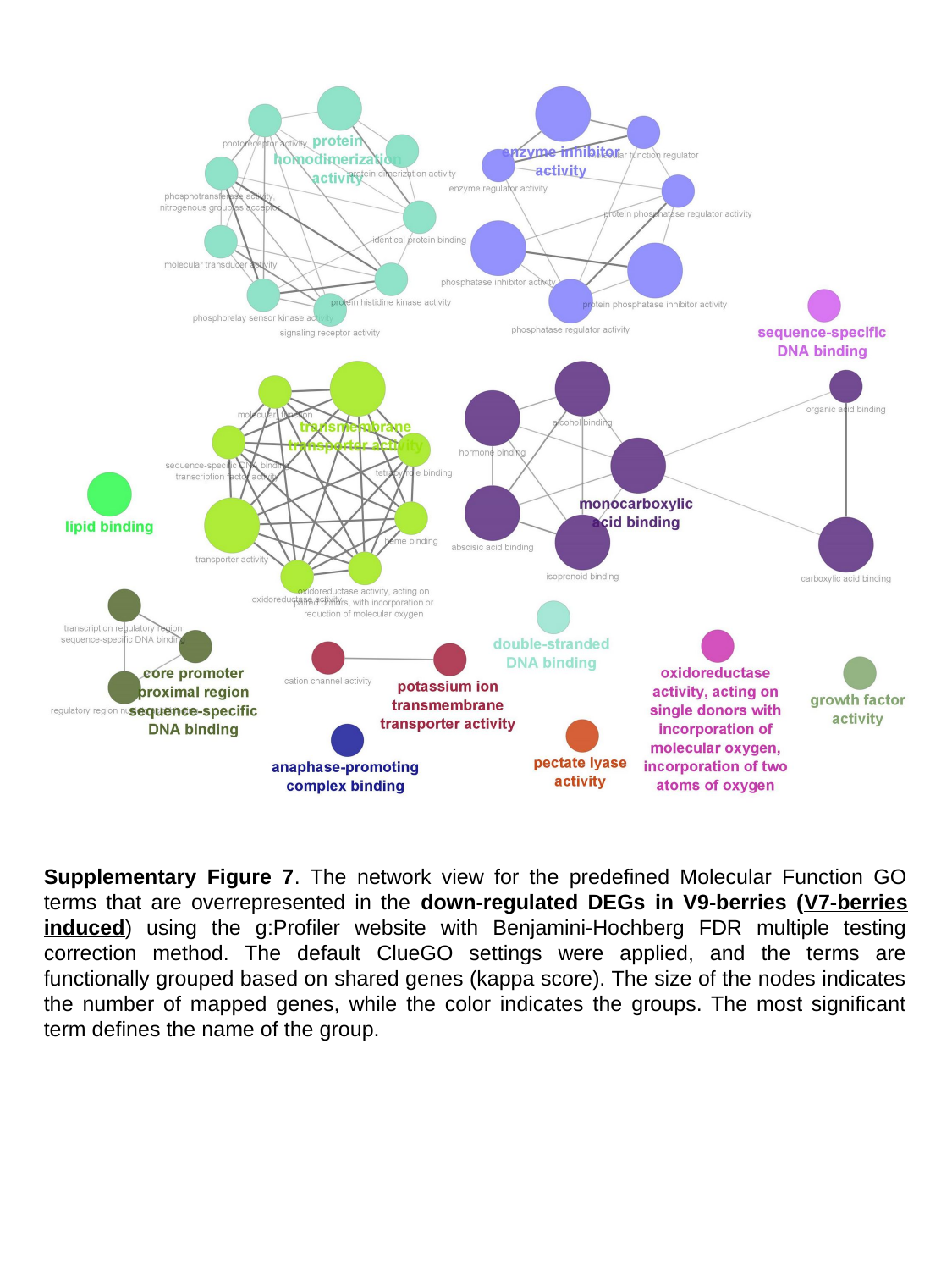

Supplementary Figure 7. The network view for the predefined Molecular Function GO terms that are overrepresented in the down-regulated DEGs in V9-berries (V7-berries induced) using the g:Profiler website with Benjamini-Hochberg FDR multiple testing correction method. The default ClueGO settings were applied, and the terms are functionally grouped based on shared genes (kappa score). The size of the nodes indicates the number of mapped genes, while the color indicates the groups. The most significant term defines the name of the group.

## Slide 8
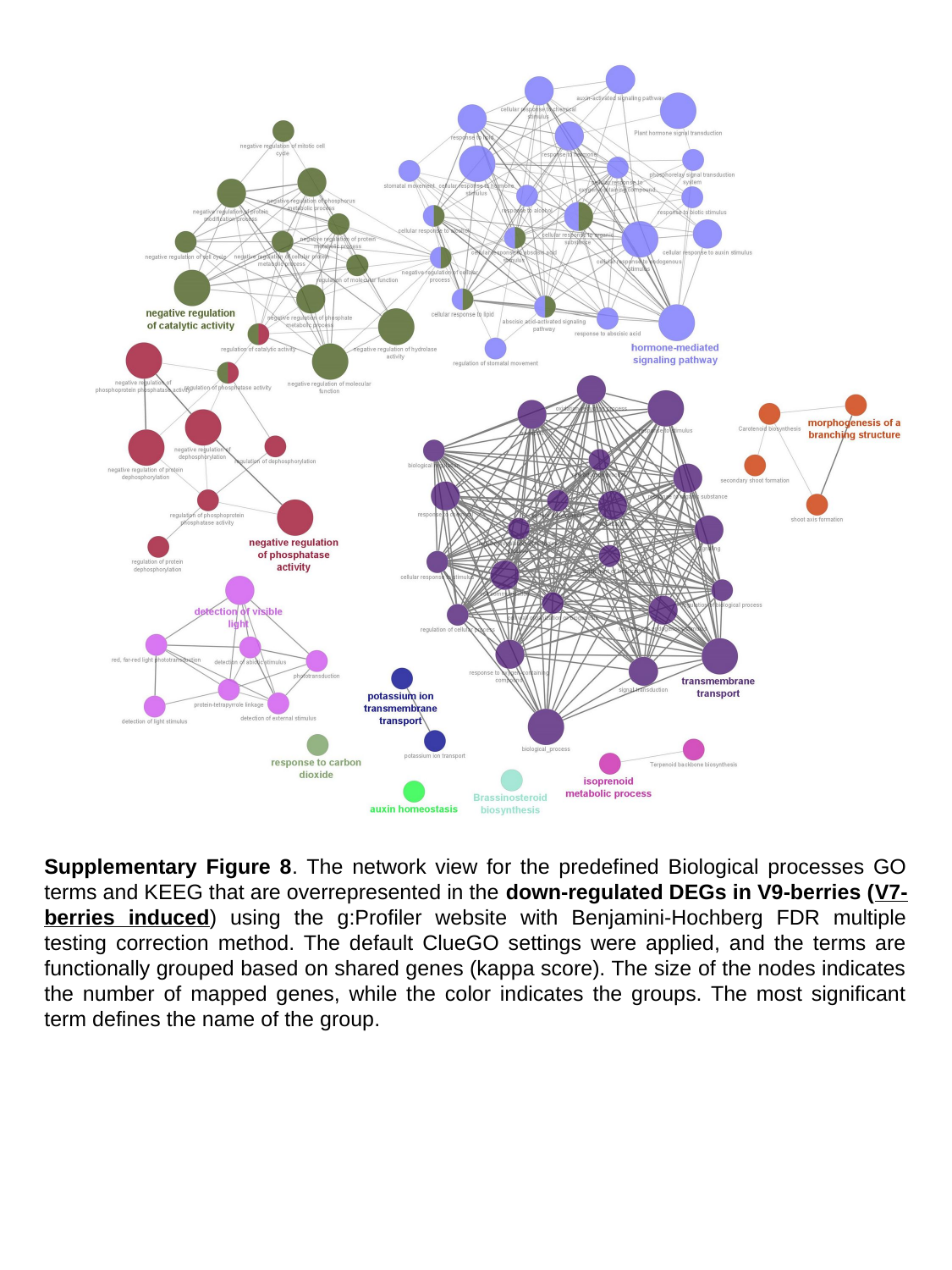

Supplementary Figure 8. The network view for the predefined Biological processes GO terms and KEEG that are overrepresented in the down-regulated DEGs in V9-berries (V7-berries induced) using the g:Profiler website with Benjamini-Hochberg FDR multiple testing correction method. The default ClueGO settings were applied, and the terms are functionally grouped based on shared genes (kappa score). The size of the nodes indicates the number of mapped genes, while the color indicates the groups. The most significant term defines the name of the group.

## Slide 9
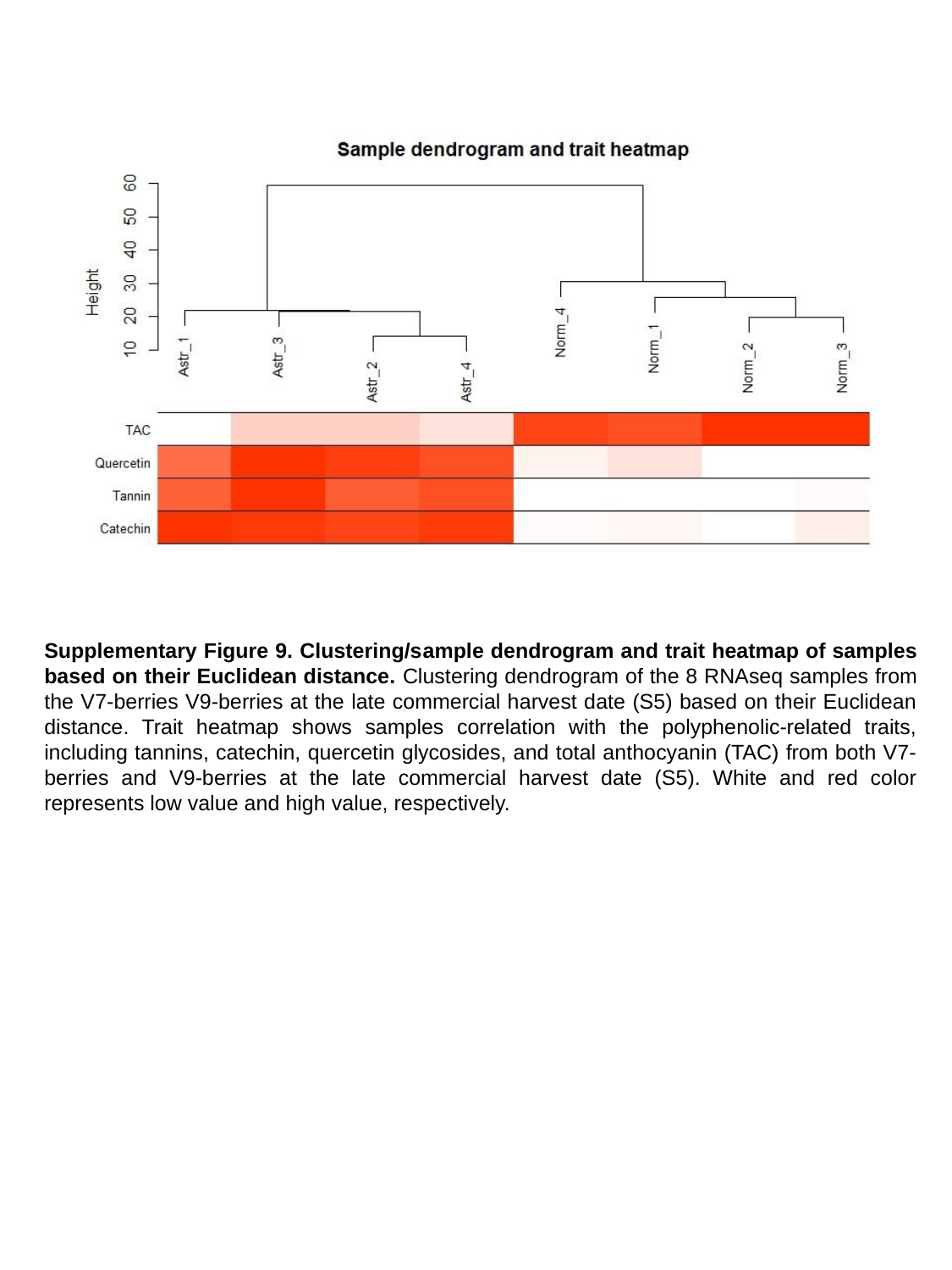

Supplementary Figure 9. Clustering/sample dendrogram and trait heatmap of samples based on their Euclidean distance. Clustering dendrogram of the 8 RNAseq samples from the V7-berries V9-berries at the late commercial harvest date (S5) based on their Euclidean distance. Trait heatmap shows samples correlation with the polyphenolic-related traits, including tannins, catechin, quercetin glycosides, and total anthocyanin (TAC) from both V7-berries and V9-berries at the late commercial harvest date (S5). White and red color represents low value and high value, respectively.

## Slide 10
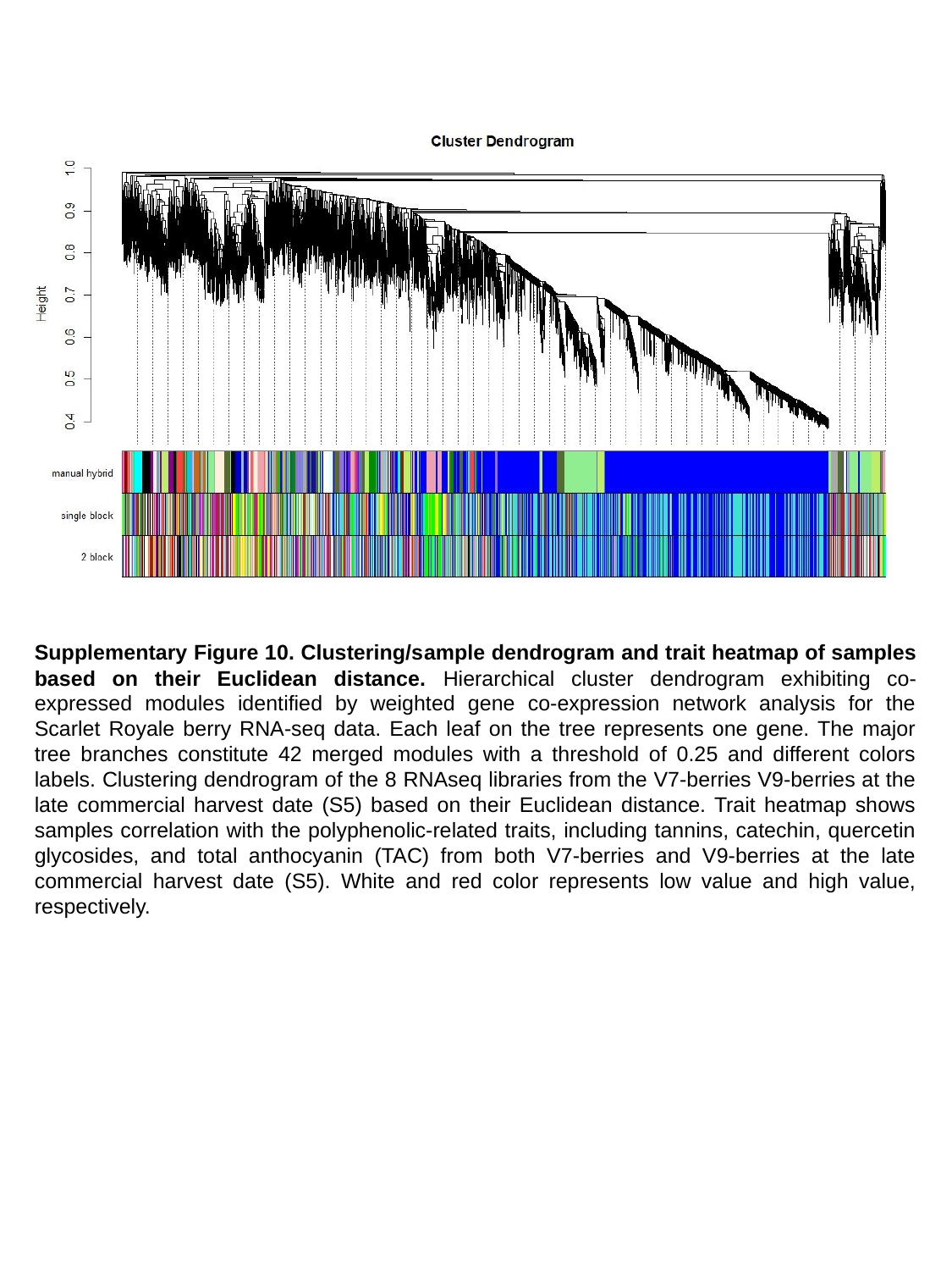

Supplementary Figure 10. Clustering/sample dendrogram and trait heatmap of samples based on their Euclidean distance. Hierarchical cluster dendrogram exhibiting co-expressed modules identified by weighted gene co-expression network analysis for the Scarlet Royale berry RNA-seq data. Each leaf on the tree represents one gene. The major tree branches constitute 42 merged modules with a threshold of 0.25 and different colors labels. Clustering dendrogram of the 8 RNAseq libraries from the V7-berries V9-berries at the late commercial harvest date (S5) based on their Euclidean distance. Trait heatmap shows samples correlation with the polyphenolic-related traits, including tannins, catechin, quercetin glycosides, and total anthocyanin (TAC) from both V7-berries and V9-berries at the late commercial harvest date (S5). White and red color represents low value and high value, respectively.

## Slide 11
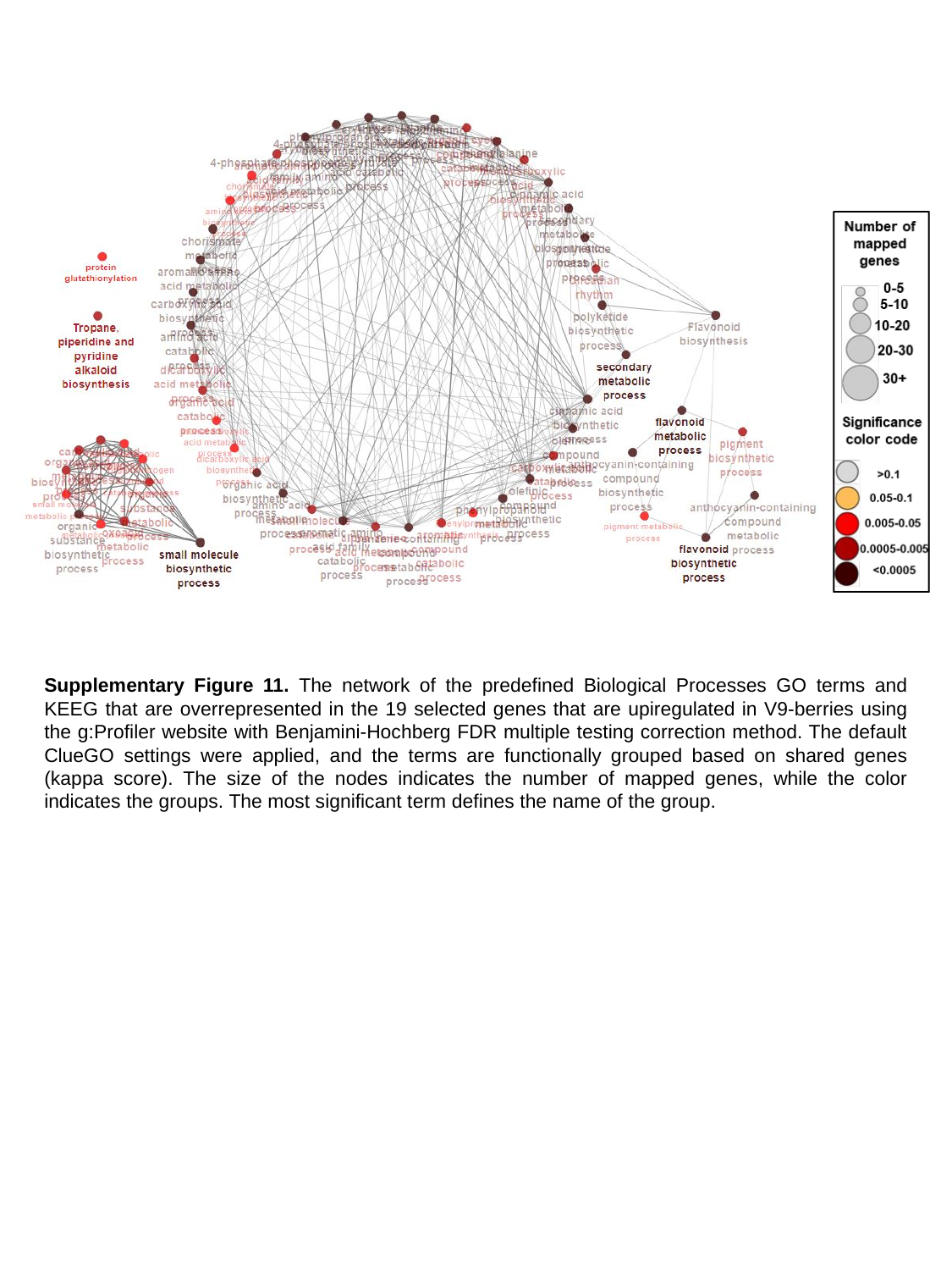

Supplementary Figure 11. The network of the predefined Biological Processes GO terms and KEEG that are overrepresented in the 19 selected genes that are upiregulated in V9-berries using the g:Profiler website with Benjamini-Hochberg FDR multiple testing correction method. The default ClueGO settings were applied, and the terms are functionally grouped based on shared genes (kappa score). The size of the nodes indicates the number of mapped genes, while the color indicates the groups. The most significant term defines the name of the group.

## Slide 12
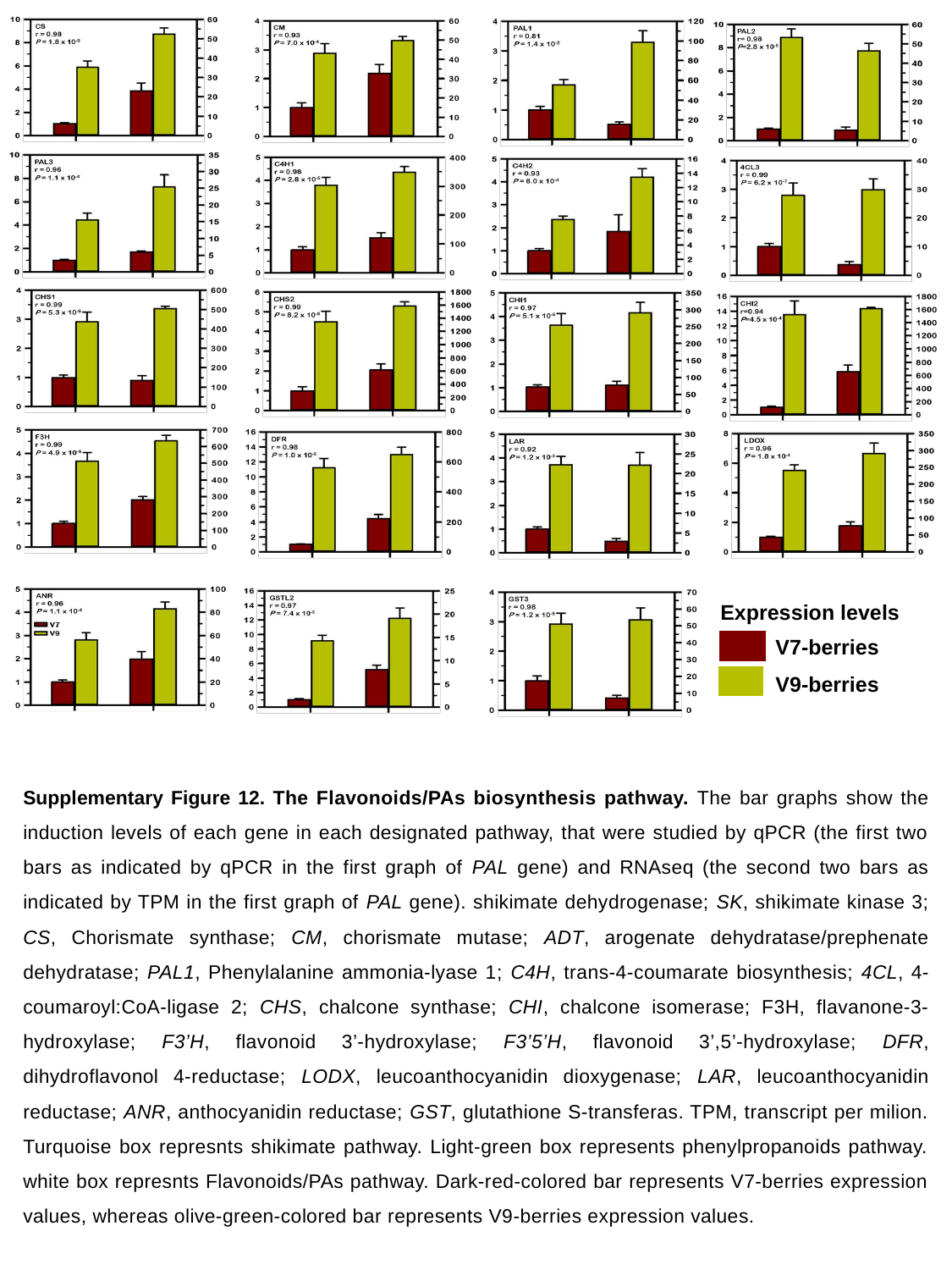

Expression levels
V7-berries
V9-berries
Supplementary Figure 12. The Flavonoids/PAs biosynthesis pathway. The bar graphs show the induction levels of each gene in each designated pathway, that were studied by qPCR (the first two bars as indicated by qPCR in the first graph of PAL gene) and RNAseq (the second two bars as indicated by TPM in the first graph of PAL gene). shikimate dehydrogenase; SK, shikimate kinase 3; CS, Chorismate synthase; CM, chorismate mutase; ADT, arogenate dehydratase/prephenate dehydratase; PAL1, Phenylalanine ammonia-lyase 1; C4H, trans-4-coumarate biosynthesis; 4CL, 4-coumaroyl:CoA-ligase 2; CHS, chalcone synthase; CHI, chalcone isomerase; F3H, flavanone-3-hydroxylase; F3’H, flavonoid 3’-hydroxylase; F3’5’H, flavonoid 3’,5’-hydroxylase; DFR, dihydroflavonol 4-reductase; LODX, leucoanthocyanidin dioxygenase; LAR, leucoanthocyanidin reductase; ANR, anthocyanidin reductase; GST, glutathione S-transferas. TPM, transcript per milion. Turquoise box represnts shikimate pathway. Light-green box represents phenylpropanoids pathway. white box represnts Flavonoids/PAs pathway. Dark-red-colored bar represents V7-berries expression values, whereas olive-green-colored bar represents V9-berries expression values.

## Slide 13
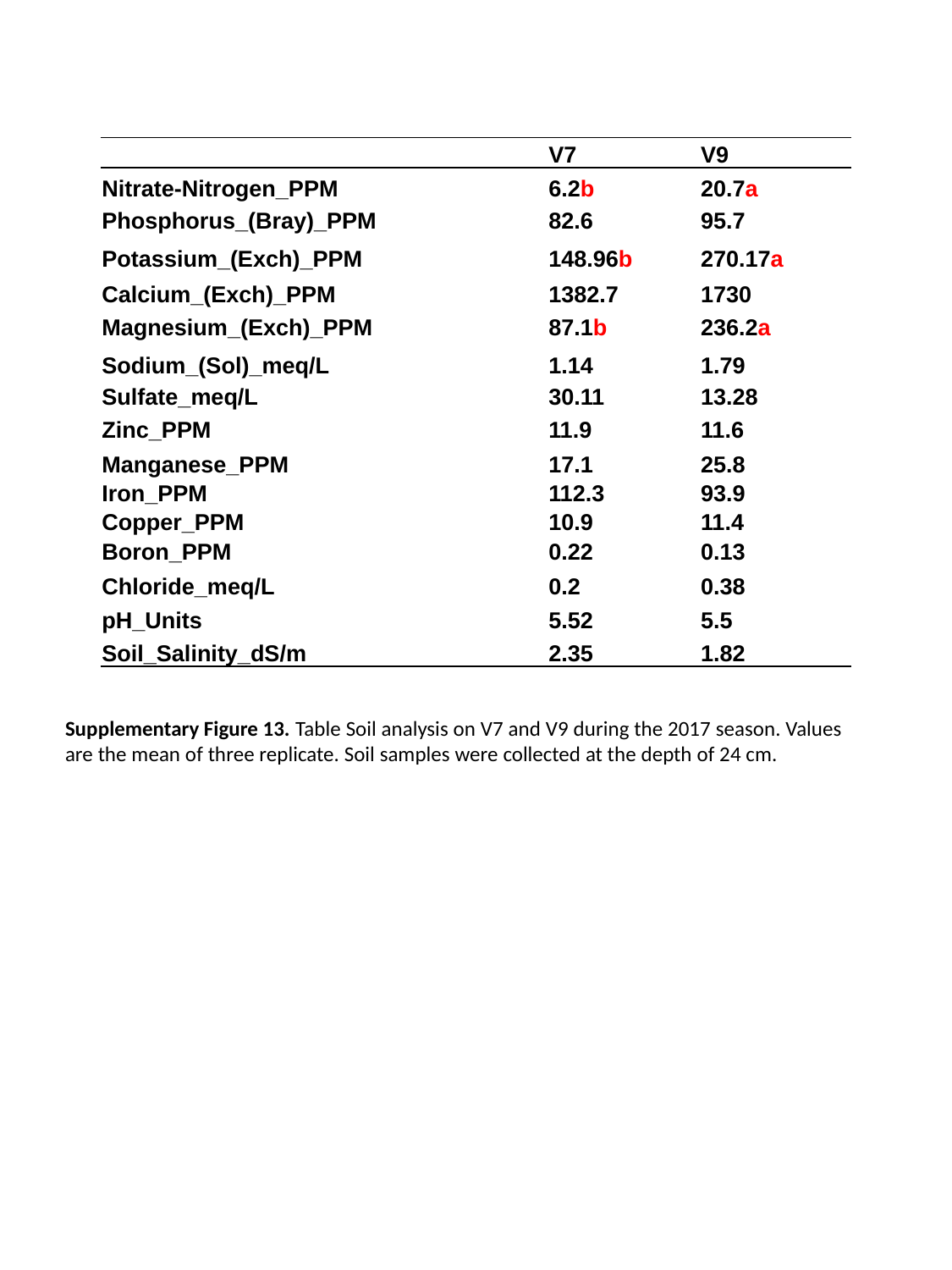

| | V7 | V9 |
| --- | --- | --- |
| Nitrate-Nitrogen\_PPM | 6.2b | 20.7a |
| Phosphorus\_(Bray)\_PPM | 82.6 | 95.7 |
| Potassium\_(Exch)\_PPM | 148.96b | 270.17a |
| Calcium\_(Exch)\_PPM | 1382.7 | 1730 |
| Magnesium\_(Exch)\_PPM | 87.1b | 236.2a |
| Sodium\_(Sol)\_meq/L | 1.14 | 1.79 |
| Sulfate\_meq/L | 30.11 | 13.28 |
| Zinc\_PPM | 11.9 | 11.6 |
| Manganese\_PPM | 17.1 | 25.8 |
| Iron\_PPM | 112.3 | 93.9 |
| Copper\_PPM | 10.9 | 11.4 |
| Boron\_PPM | 0.22 | 0.13 |
| Chloride\_meq/L | 0.2 | 0.38 |
| pH\_Units | 5.52 | 5.5 |
| Soil\_Salinity\_dS/m | 2.35 | 1.82 |
Supplementary Figure 13. Table Soil analysis on V7 and V9 during the 2017 season. Values are the mean of three replicate. Soil samples were collected at the depth of 24 cm.

## Slide 14
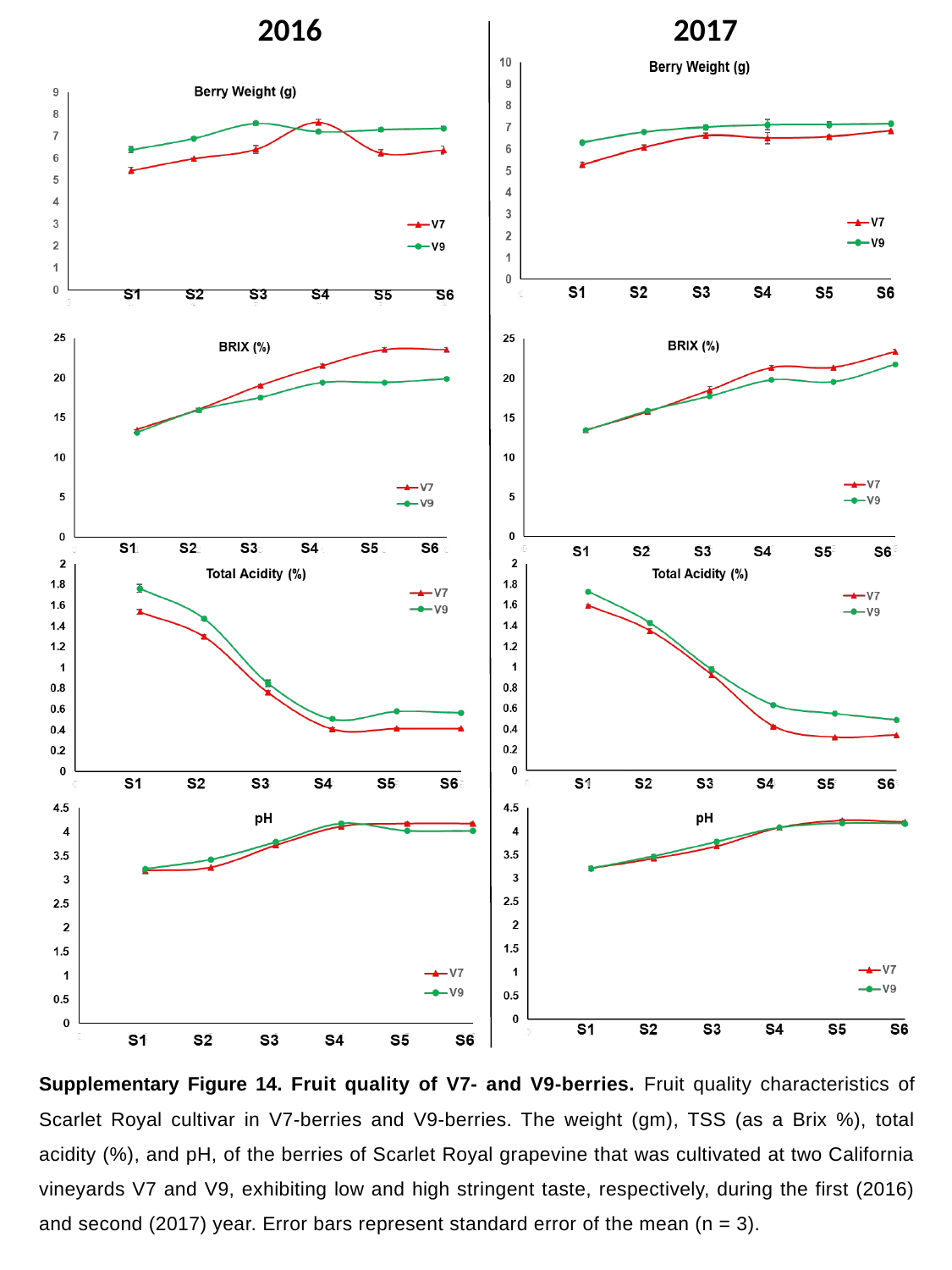

2016 2017
Supplementary Figure 14. Fruit quality of V7- and V9-berries. Fruit quality characteristics of Scarlet Royal cultivar in V7-berries and V9-berries. The weight (gm), TSS (as a Brix %), total acidity (%), and pH, of the berries of Scarlet Royal grapevine that was cultivated at two California vineyards V7 and V9, exhibiting low and high stringent taste, respectively, during the first (2016) and second (2017) year. Error bars represent standard error of the mean (n = 3).
